# Analysis of gelsolin expression pattern in developing chicken embryo reveals high *GSN* expression level in tissues of neural crest origin

**DOI:** 10.1007/s00429-014-0923-5

**Published:** 2014-10-29

**Authors:** Antonina Joanna Mazur, Gabriela Morosan-Puopolo, Aleksandra Makowiecka, Maria Malicka-Błaszkiewicz, Dorota Nowak, Beate Brand-Saberi

**Affiliations:** 1 Department of Cell Pathology, Faculty of Biotechnology, University of Wroclaw, ul. Joliot-Curie 14a, 50-383 Wrocław, Poland; 2 Department of Anatomy and Molecular Embryology, Ruhr University of Bochum, Bochum, Germany

**Keywords:** Gelsolin, Chicken development, Nervous system, Neural crest, Optic and olfactory systems, Brain

## Abstract

Gelsolin is one of the most intensively studied actin-binding proteins. However, in the literature comprehensive studies of *GSN* expression during development have not been performed yet in all model organisms. In zebrafish, gelsolin is a dorsalizing factor that modulates bone morphogenetic proteins signaling pathways, whereas knockout of the gelsolin coding gene, *GSN* is not lethal in murine model. To study the role of gelsolin in development of higher vertebrates, it is crucial to estimate *GSN* expression pattern during development. Here, we examined *GSN* expression in the developing chicken embryo. We applied numerous methods to track *GSN* expression in developing embryos at mRNA and protein level. We noted a characteristic *GSN* expression pattern. Although *GSN* transcripts were present in several cell types starting from early developmental stages, a relatively high *GSN* expression was observed in eye, brain vesicles, midbrain, neural tube, heart tube, and splanchnic mesoderm. In older embryos, we observed a high *GSN* expression in the cranial ganglia and dorsal root ganglia. A detailed analysis of 10-day-old chicken embryos revealed high amounts of gelsolin especially within the head region: in the olfactory and optic systems, meninges, nerves, muscles, presumptive pituitary gland, and pericytes, but not oligodendrocytes in the brain. Obtained results suggest that *GSN* is expressed at high levels in some tissues of ectodermal origin including all neural crest derivatives. Additionally, we describe that silencing of *GSN* expression in brain vesicles leads to altered morphology of the mesencephalon. This implies gelsolin is crucial for chicken brain development.

**Electronic supplementary material:**

The online version of this article (doi:10.1007/s00429-014-0923-5) contains supplementary material, which is available to authorized users.

## Introduction

Actins, abundantly expressed in all animal cell types, are capable of forming polymers and take part in several cellular processes such as cell motility, chemoattractant-controlled (or directed) migration, trafficking of cellular organelles and chromosomes, junction formation, mitosis, transcription, and muscle contraction (Perrin and Ervasti 2010). Consequently, it is understandable that actin dynamics have to be strictly controlled. There are more than 100 Actin-binding proteins (ABPs), which regulate actin polymerization/depolymerization and involvement in several cellular processes (Winder and Ayscough 2005).

Although it was shown that actin is indispensable for mammalian embryonic development (Shawlot et al. 1998), not much is known about the role of ABPs in vertebrate embryo development. There are, however, some exceptions. For instance, it has been shown that thymosin beta4 plays a role in brain development of chicken embryos (Wirsching et al. 2012) and that it is expressed in the neural tube and brain vesicles, the heart, blood vessels, and feathers (Dathe and Brand-Saberi 2004). The presence of thymosin beta4 mRNA was strongly manifested in neural tissues like the neural tube and dorsal root ganglia. These results are similar to those obtained by researchers, who studied thymosin beta4 expression in mouse embryos (Gómez-Márquez et al. 1996). In contrast, another thymosin beta family member, thymosin beta15^avian^ is asymmetrically expressed in Hensen’s node and could thus be involved in left/right axis formation; it has been described to have a function in myogenesis in chicken embryos (Chankiewitz et al. 2014). Other G-actin-binding proteins belong to the ADF/cofilin family. Actin depolymerizing factor (ADF) seems to be dispensable during mouse embryo development (Gurniak et al. 2005), but n-cofilin is crucial for migration of cells derived from the paraxial mesoderm. On the other hand, decreased expression of non-muscle cofilin (n-cofilin) in murine preimplantation embryos is important for compaction during blastocyst formation (Ma et al. 2009).

Here, we focus on the gelsolin expression pattern in developing chicken embryos. Gelsolin is coded by one gene (*GSN*); however, its expression yields several protein isoforms. In humans, the existence of three isoforms: plasma (isoform a, 86 kDa), cytoplasmic (isoform b, 81 kDa), and “brain” (isoform c, 82 kDa) is well documented. Furthermore, the presence of some other isoforms can be predicted. Gelsolin, a Ca^2+^, phosphatidylinositol 4,5-biphosphate (PIP_2_) and pH-dependent six-domain (G1–G6) protein, severs actin filaments, caps the barbed ends of actin filaments and under certain conditions nucleates actin monomers (Mannherz et al. 2010; Li et al. 2012; Nag et al. 2013). However, gelsolin seems to have more functions than severing actin filaments in the cells or scavenging microfilaments in the body fluids. Gelsolin is also directly involved in nuclear processes such as transcription. It binds to hypoxia-inducible factor 1 (HIF-1), a transcription factor (Li et al. 2009) and forms complexes with steroid hormones, e.g., androgens (Nishimura et al. 2003) and estrogens (Ambrosino et al. 2010). This process is crucial for entry of hormones bound to their receptor into the nucleus and for forming transcription complexes. Moreover, several reports point toward involvement of gelsolin in various human cancers, due to the observation that *GSN* expression was downregulated in numerous types of cancers, e.g., breast, bladder, colon, gastric, kidney, lung, oral, ovarian, pancreatic, and prostate cancer (Li et al. 2012). This is corroborated by results published by Tanaka et al. (2006) showing that downregulation of *GSN* expression by applying siRNA promoted epithelial–mesenchymal transition (EMT) of mammary epithelial cells. It suggests gelsolin is an important tumor suppressor (Li et al. 2012).

Our previous studies focused on the role of gelsolin in cell motility of human tumor cells (Mazur et al. 2010) with special emphasis on colon adenocarcinoma (Litwin et al. 2009, 2012) and melanoma cells (Litwin et al. 2012). Since gelsolin probably plays a significant role in cell transformation into tumor cells, it is likely to have a role during embryonic development, because in both situations cells detach, migrate/invade and colonize new environments. That is why we decided to determine *GSN* expression pattern in chicken embryo development, since not much is known about the role of gelsolin in vertebrate embryo development. Kwiatkowski’s group generated mice with *GSN* gene knockout, which were subsequently subjected to several studies. They reported that the absence of gelsolin was not lethal and gave relatively mild symptoms including impaired fibroblast motility and inflammatory response (Witke et al. 1995), or that it affected the morphogenesis of mammary ducts (Crowley et al. 2000). It could be explained by taking over of gelsolin functions in *GSN* ^−*/*−^ mice by other members of the gelsolin protein family which in higher vertebrates includes CapG, adseverin, flightless I, advillin, villin, villin-like protein, and supervillin (Nag et al. 2013). Interestingly, it was shown that gelsolin is a dorsalizing factor in *Danio rerio* development. Depleting embryos of *GSN* mRNA resulted in affected development of head structures and eye and resulted in less pigmented embryos. Kanungo et al. (2003) showed that gelsolin has an important regulatory function as a modulator of bone morphogenetic protein signaling pathways involving chordin which is responsible for the formation of the dorsoventral axis in zebrafish. In this work, we present the first comprehensive study of *GSN* expression in the chicken embryo, which answers questions at which developmental stages *GSN* is expressed, what is the pattern of expression and if gelsolin is present at protein level in chicken. Additionally, we show that gelsolin is required for early brain development.

## Materials and methods

### Ethics statement

Regulations concerning the usage of chicken embryos for experimental procedures are described elsewhere (Morosan-Puopolo et al. 2014). The chicken embryos sacrificed for this work were between developmental stages HH8 and HH36 (E 10). No permits were required for this study.

### Bioinformatics analysis

Amino acid sequence alignments were performed using MAFFT, the multiple alignment program for amino acid or nucleotide sequences, version 7 (Katoh et al. 2005; Katoh and Standley 2013). Following amino acid sequences were analyzed: human gelsolin isoforms a (NP_000168.1, Pubmed), b (Pubmed: NP_937895.1), c (Pubmed: NP_001121138.1), d (Pubmed: NP_001244958.1), f (Pubmed: NP_001121135.2), and chicken gelsolin (NP_990265.1, Pubmed). For other analyses following gene sequence was used: chicken (*Gallus domesticus*) *GSN*: 395774 (Pubmed).

### Cloning

Dermis of chicken embryo at stage HH36 (E10) was dissected, followed by rinsing in ice-cold PBS, next homogenized by cutting and frozen at −80 °C. After tissue thawing total RNA was isolated with the help of NucleoSpin^®^ RNA II Kit (Macherey–Nagel). 1 μg of RNA was reverse transcribed using High Capacity cDNA Reverse Transcription Kit (Applied Biosystems) following the manufacturer’s instructions. PCR reactions with following primers 5′-CTGGTTAGGAGATGAAAGCTC-3′ and 5′-CTGGTCCTTGTCCCTCCAGTTC-3′ for gelsolin were carried out to achieve product, which was subsequently cloned into pDrive plasmid (Qiagen). Primers were designed based on a nucleotide sequence coding for chicken gelsolin found in Pubmed under NM_204934 number. A *Pyrococcus furiosus* (*Pfu*) polymerase was used (Fermentas) to avoid introducing any mistakes into nucleotide sequence. The correctness of DNA construct was verified by sequencing.

### RNA interference

The strategy for obtaining plasmids coding for shRNAmirs targeting gelsolin mRNA was based on *Tol2* transposon-mediated technique (Iguchi et al. 2012). The Tol2-EGFP vector system (Koga et al. 1996; Kawakami et al. 2000; Kawakami 2007; Sato et al. 2007) was kindly provided as a gift from Koichi Kawakami (Division of Molecular and Developmental Biology, National Institute of Genetics, Mishima, Shizuoka 411-8540, Japan). Following oligonucleotides were ordered: for shRNAmir-*Gd*_*GSN*_921, 5′-TGCTGTTGACAGTGAGCGCACTGATACAGCCAATAGAAAGTAGTGAAGCCACAGATGTACTTTCTATTGGCTGTATCAGTTTGCCTACTGCCTCGGA-3′; for shRNAmir-*Gd*_*GSN*_1137, 5′-TGCTGTTGACAGTGAGCGCGCTACTGATTTCATTGATAAGTAGTGAAGCCACAGATGTACTTATCAATGAAATCAGTAGCTTGCCTACTGCCTCGGA-3′; for shRNAmir-*Gd*_*GSN*_1647, 5′-TGCTGTTGACAGTGAGCGACCACCTC-ATCTGATGAGCATGTAGTGAAGCCACAGATGTACATGCTCATCAGATGAGGTGGCTGCCTACTGCCTCGGA-3′; for scRNAmir-*Gd*_*GSN*_921, 5′-TGCTGTTGACA-GTGAGCGCGAATACGGATAAGCATACACATAGTGAAGCCACAGATGTATGTGTATGCTTATCCGTATTCTTGCCTACTGCCTCGGA-3′; for scRNAmir-*Gd*_*GSN*_1137, 5′-TGCTGTTGACAGTGAGCGCTTATTAATACCGGTATGACGTTAGTGAAGCCACAG-ATGTAACGTCATACCGGTATTAATAATTGCCTACTGCCTCGGA-3′; for scRNAmir-*Gd*_*GSN*_1647, 5′-TGCTGTTGACAGTGAGCGCTTCGCCAGGCGACCATTATGCTAGTGAAGCCACAGATGTAGCATAATGGTCGCCTGGCGAATTGCCTACTGCCTCGGA-3′. The sequences, which are underlined correspond to antisense and sense target sequences based on a nucleotide sequence coding for chicken gelsolin found in Pubmed under NM_204934 number. Primers: 5′-GATGGCTGCTCGAGAAGGTATATTGCTGTTGAC-AGTGAGCG-3′ and 5′-GTCTAGA-GGAATTCCGAGGCAGTAGGCA-3′ were used to amplify by PCR mir-based scRNAs and shRNAs. A HotStarTaq^®^ DNA polymerase with provided Q-Solution^®^ was used (Qiagen) to amplify templates with high degree of secondary structures. PCR products were cloned into pT2K-TBI-shRNAmir vector using *Xho*I and *EcoR*I sites. The correctness of DNA constructs was verified by sequencing.

### In ovo electroporation

Electroporation was performed according to the procedure described elsewhere (Scaal et al. 2004; Dai et al. 2005) using the Intracel TSS20 OVODYNE device. 1–2 μl of plasmids solution (2–4 μg/μl) was electroporated into the brain vesicles of embryos at HH-stages 11–12. The plasmids were mixed in the following ratio: pT2K-CAGGS-rtTA-M2 (rtTA-M2 doxycycline-binding element): pT2K-TBI-shRNAmirs: pCAGGS-T2TP (*Tol2* transposase) = 1:2:2 (Iguchi et al. 2012). After electroporation, the eggs were sealed with tape and reincubated. 12 h later 500 μl of doxycycline (Dox) dissolved in HANKS solution (140 mM NaCl, 5.4 mM KCl, 5.6 mM glucose, 0.34 mM Na_2_HPO_4_, 10 mM HEPES, 1 mM CaCl_2_, pH 7.0) to final concentration of 0.1 mg/ml was injected between the embryo and yolk to trigger the expression of scRNAmirs or shRNAmirs and EGFP (Watanabe et al. 2007). 24 h later the embryos were in ovo photographed and harvested for following in situ hybridization experiments.

### In situ hybridization

Fertilized chicken eggs were incubated at 37 °C and 72 % humidity and staged according to Hamburger and Hamilton (1992). Whole mount chicken embryos at HH-stages 9–36 were fixed overnight at 4 °C in 4 % formaldehyde (FA). The detailed protocol is described elsewhere (Nieto et al. 1996). As a template for preparing riboprobes pDrive-gelsolin fragment plasmid was used. Two probes were prepared, sense and antisense. Both probes were digoxigenin labeled with DIG RNA Labeling Mix (Roche) according to manufacturer’s protocols. Sense probe being a negative control gave no signal. The probe recognizing *CECR2* transcript is described elsewhere (Chen et al.). Selected stained embryos were embedded in 3 % agarose and cut with the help of Leica VT 1000S vibratome at 60 μm, next mounted on glass and finally analyzed using Axioplan 2 microscope (Zeiss) and Axio Vision software (Zeiss).

### RT-PCR analysis

Embryos at different stages and organs dissected from embryos at stage HH36 were stored in RNA*later* RNA stabilization reagent (QIAgen) at −20 °C. Following total RNA was isolated with the help of NucleoSpin^®^ RNA II Kit (Macherey–Nagel). 0.5 μg of RNA was reversely transcribed using High Capacity cDNA Reverse Transcription Kit (Applied Biosystems) following the manufacturer’s instructions. 1 μl of transcribed cDNA was used for the subsequent PCR with primers listed in Table 1, the reaction final volume was 15 μl, PCRs were carried out under following conditions: initial denaturation 2 min 95 °C, 30 cycles of denaturation (30 s at 95 °C), annealing (30 s at Tm) and elongation (1 min at 72 °C). As reference gene served chicken *ACTB.* PCR products were subjected to horizontal DNA electrophoresis in 2 % agarose gel/1 × Tris-acetate-EDTA (TAE) buffer, as a molecular mass marker served GeneRuler™ 50 bp DNA ladder (Fermentas). Pictures were taken with ChemiDoc™ MP System (Bio-Rad) and further analyzed with the help of ImageLab 4.0 software (Bio-Rad).

**Table 1:** Nucleotide sequences, amplicon sizes, and annealing temperatures (Tm) of primers used in RT-PCR analysis

Primer	Sequence	Amplicon size (nt)	Tm (°C)
*Gd* *_ACTB*_f	5′-CAGAAGGAGATCACAGCCCTG-3′	221	63
*Gd* *_ACTB*_r	5′-CCAACACCCACACCCCTGTG-3′		
*Gd*_*GSN*_f	5′-GCCATGAATCCTCAACGTTC-3′	215	63
*Gd*_*GSN*_r	5′-CATCCTTGACCTTGGCAGT-3′		

### Isolation of tissue extracts

For Western blot analysis, whole chicken embryos at HH-stages 9–25 and chosen organs from chicken embryos at stage HH36 were lysed on ice with RIPA buffer (50 mM Tris HCl pH 8.0, 150 mM NaCl, 1 % NP-40, 0.5 % sodium deoxycholate, 0.1 % SDS) supplemented with proteases inhibitors cocktail (PIC) (Sigma) diluted at 1:100. Next the lysates were threefold frozen-thawed and centrifuged at 10,000×*g* for 10 min at 4 °C. Supernatants for further analysis were stored at −80 °C.

### Western blot analysis

Protein concentration in cellular extracts was determined by the standard Bradford procedure (Bradford 1976). Samples of identical protein amount (30 µg) were separated by 10 % polyacrylamide gel electrophoresis in the presence of sodium dodecylsulfate (SDS-PAGE) according to Laemmli (1970), as a molecular mass marker served PageRuler™ Prestained Protein Ladder (Fermentas). This was followed by transfer to nitrocellulose membrane, using the procedure described elsewhere (Towbin et al. 1979). Monoclonal rabbit anti-gelsolin antibodies (Abcam, clone EPR1942) at dilution 1:5,000 were used to visualize gelsolin band on nitrocellulose. β actin and β tubulin recognized by monoclonal mouse anti-β actin (Sigma, clone AC-15) and anti-β tubulin (TUB 2.1) antibodies, were used as reference proteins. Secondary antibodies conjugated to horseradish peroxidase (HRP) were applied according to the manufacturer’s protocols (Cell Signaling). Immunoblots were developed using the Western blotting Luminol Reagent (Santa Cruz Biotechnology), photos of blots were taken with ChemiDoc™ MP System (Bio-Rad) and further analyzed with the help of ImageLab 4.0 software (Bio-Rad).

### Immunohistochemistry

Whole embryos at stage HH33 and parts of embryos at stage HH36 were collected and fixed in 4 % formaldehyde (FA) for at least 24 h. Next samples were dehydrated and embedded in Paraplast^®^ (Sigma), 7 μm thick sections were cut with the help of a rotation microtome (Leitz) and mounted on SuperFrost Plus microscope slides (Menzel-Gläser). FA-fixed and paraffin-embedded sections of chicken embryos were deparaffinized in xylene and subsequently rehydrated by standard procedure. Antigen retrieval was performed by boiling slides in citrate buffer (10 mM Na-citrate pH 6.0, 0.05 % Tween 20) for 20 min. Endogenous peroxidase activity was blocked according to EnVision™ System-HRP (AEC)^+^ kit manufacturer’s protocol (DAKO). Sections were blocked for 1 h with 3 % goat serum in 1 % bovine serum albumin (BSA) in 50 mM Tris pH 7.6. Following the slides were incubated overnight at 4 °C with primary rabbit anti-gelsolin antibodies (Abcam, clone EPR1942) diluted at 1:500. As a negative control normal rabbit antibodies were used. DAKO EnVision™ System-HRP (AEC)^+^ kit was used to visualize the sites recognized by primary antibodies. The reaction was developed with 3-amino-9 ethylocarbazol (AEC) for 10 min. Next, the sections were counterstained with Mayer’s hematoxylin solution (Sigma) for 10 min to visualize cell nuclei. Photos at lower magnifications were taken using Olympus SZ61 stereo microscope and photos at higher magnification were taken using Olympus FV500 microscope.

## Results

### The chicken gelsolin sequence

**Fig. 1:**
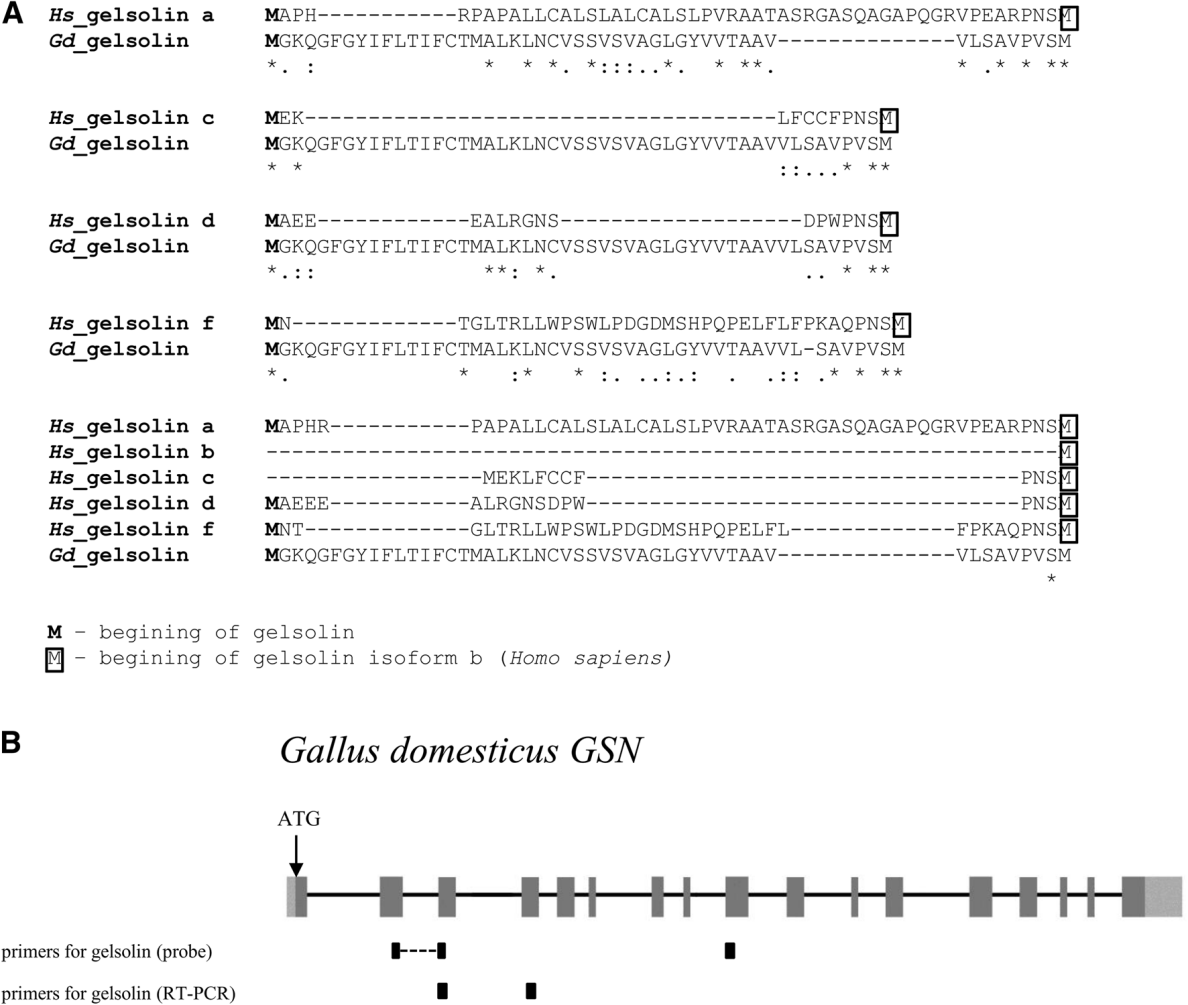
Analysis of amino acid sequence of chicken gelsolin and molecular biological strategies for probe preparation and RT-PCR analysis. **a** Alignments of N-termini of known and presumptive human gelsolin isoforms and chicken gelsolin precursor. Human gelsolin isoform b known as cytoplasmic gelsolin does not possess an additional amino acid sequence at N-terminus. Thus, it was not subjected to this analysis. In cases of other human gelsolin isoforms, the length of additional amino acid sequence varies with the longest one for gelsolin isoform a described as plasma gelsolin. Amino acid sequences alignments were done with the help of the MAFFT program. “*Asterisk*” means identical, “*colon*” represents conserved substitutions, “*dot*” means semi-conserved substitution. **b** Schematic map of chicken gene coding for gelsolin precursor (gene id: 395774, Pubmed). *Gray boxes* represent exons and pale *gray boxes* reflect UTRs. Sites, to which primers used for riboprobes preparation and for RT-PCR analysis anneal, are shown schematically as *bold bars*. Note that the probe recognizing gelsolin spans exons 2–9. Primers for RT-PCR reaction detecting gelsolin span exons 3–4

According to information about the chicken gelsolin gene (*GSN*, gene id: 395774, Pubmed), there is only one transcript of this gene coding for chicken gelsolin, of which the amino acid sequence is 77 % identical to the human gelsolin isoform a—known as plasma gelsolin (Online Resource 1, Fig. S1A). The human isoform b of gelsolin, called cytoplasmic gelsolin ubiquitously present in numerous cell types is a shorter protein in comparison to human plasma gelsolin. In Online Resource 1, Fig. S1A, the first amino acids of both plasma and cytoplasmic gelsolins are marked. Major differences between chicken and human gelsolin are localized in the N-terminus of the compared amino acid sequences. Because in *Homo sapiens*, there is also the gelsolin isoform c found in oligodendrocytes of the central nervous system (CNS) (Vouyiouklis and Brophy 1997) and possibly other isoforms are also present (Nag et al. 2013), we additionally compared the N-terminus of chicken gelsolin with the N-termini of human gelsolin isoforms c, d, and f (Fig. 1a). Isoforms of human gelsolins differ only within their N-termini. The bioinformatical analysis revealed that the N-terminus of chicken gelsolin is not explicitly similar to any human gelsolin isoform. We have prepared a plasmid coding for two probes, sense and antisense, which hybridize to the *GSN* transcript (Fig. 1b). After cloning of cDNA fragment coding part of chicken gelsolin into pDrive plasmid the DNA construct was sequenced to check the correctness of cloning. It came out there were two discrepancies in positions 743 and 786 (counting from ATG codon of plasma gelsolin) of nucleotide sequence comparing to nucleotide sequence found in Pubmed under NP_990265.1 number. Because of that we decided to clone the whole cDNA coding for gelsolin and sequence it. For this purpose, we took Phusion™ polymerase, which ensures high-fidelity amplification. It came out there were some differences between sequenced DNA and nucleotide sequence (NM_204934) found in Pubmed. Most of them do not result in changed amino acids. However, four amino acids are different (Online Resource 1, Fig. S1A). Instead of isoleucine in position 13 there is valine, in position 98, there is lysine instead of arginine. In position 248, there is arginine and not lysine, and in position 661, there is histidine instead of tyrosine. Interestingly, in corresponding to 248 and 661 positions in human gelsolin, there are arginine (position 252) and histidine (position 665), respectively. Differing amino acids are located in signal peptide (Val^13^), domain G1 (Lys^98^), domain G2 (Arg^248^), and in the linker region between domains G5 and G6 (His^661^) (Online Resource 1, Fig. S1B). Any of these residues plays a special role in functioning of gelsolin (Online Resource 1, Fig. S1B) (Choe et al. 2002; Nag et al. 2009). The nucleotide sequence found in Pubmed under the number: NM_204934 was derived from sequence published under following number: AF042795.1 and has not been yet verified by NCBI, what could explain the differences between this sequence and sequence coming from our analysis.

### Whole mount in situ hybridization experiments

**Fig. 2:**
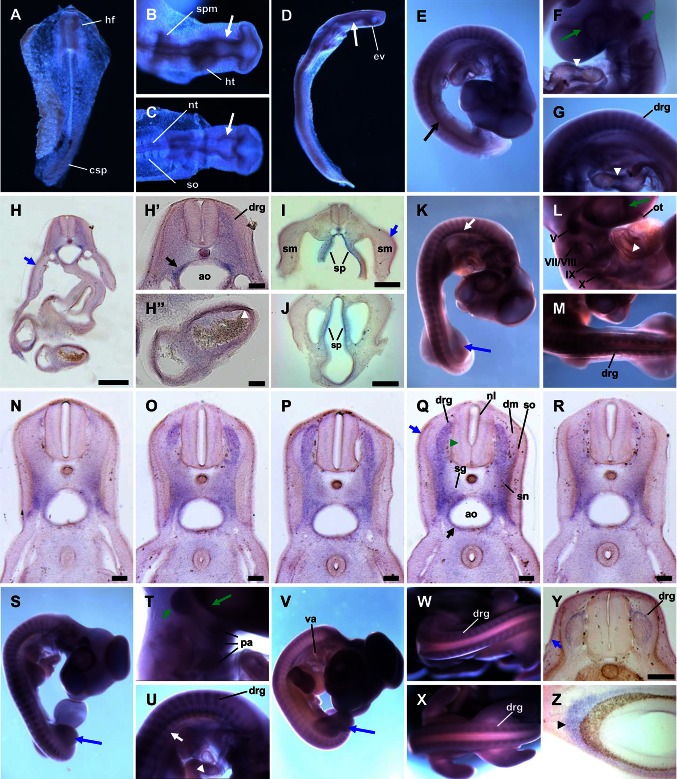
In situ hybridization experiments with the probe recognizing gelsolin transcript to whole mount chicken embryos at early stages. **a**–**g**, **k**–**m**, **s**–**x** Whole mount in situ hybridization; **h**–**j**, **n**–**r**, **y**, **z**: vibratome sections. **a** Stage HH8 embryo showing expression of *GSN* at a relatively low level and uniformly distributed within embryo with stronger expression in the head folds and the caudal segmental plate. **b**–**d** Stage HH10–11 embryo. Distinct expression of *GSN* is observed in the eye and brain vesicles (*long white arrows*), the midbrain, the heart tube, the splanchnic mesoderm, and the neural tube. Note the absence of *GSN* expression in the somites. **e**–**g** Stage HH18 embryo. *GSN* is highly expressed and predominantly present in the head mesenchyme, the eye cup (*long green arrow*), the atrioventricular canal (*white arrowheads*), cranial ganglia (*short green arrow*), dorsal root ganglia, and the splanchnopleure (*long black arrow*). **h**–**j** Vibratome sections of the embryo shown in **e**–**g**. **h′**–**h″** Higher magnifications of **h**. Note prominent *GSN* expression in a layer of mesenchymal cells surrounding aorta (*short black arrow*), the atrioventricular canal (*white arrowhead*), the splanchnopleure, and dermal cells underlying the epidermal ectoderm (*short blue arrows*). Note absence of signal in blood cells and the somatopleure. **k**–**m** Stage HH21 embryo. Expression pattern of *GSN* is significant and again high expression level is observed in the olfactory tract, cranial ganglia (*V* trigeminal, *VII* geniculate, *VIII* vestibular acoustic, *IX* petrosal, and *X* nodose ganglia), dorsal root ganglia, the sympathetic trunk (*short white arrow*), the eye cup (*long green arrow*), the atrioventricular canal (*white arrowhead*), and limbs mesenchyme (*long blue arrow*). **n**–**r** Serial vibratome sections of embryo showed in **k**–**m**. Note strong *GSN* expression in dorsal root ganglia, the spinal nerve, spinal ganglia, mesenchymal cells surrounding aorta (*short black arrow*), the neuroepithelial layer of neural tube, the mantle layer of neural tube (*green arrowhead*), and dermal cells underlying the epidermal ectoderm (*short blue arrow*). **s**–**u** Stage HH22 embryo. *GSN* higher expression levels are found especially in pharyngeal arches, the eye (*long green arrow*), cranial ganglia (*short green arrow*), dorsal root ganglia, the sympathetic trunk (*white short arrow*), the atrioventricular canal (*white arrowhead*), and limbs mesenchyme (*long blue arrow*). **v**–**x** Stage HH25 embryo. Relatively high *GSN* expression is observed in the head, the eye, within limbs mesenchyme (*long blue arrow*), visceral arches and dorsal root ganglia, which staining pattern in the posterior part of embryo (**x**) is different in comparison to the proximal part of embryo (**w**). **y**, **z** Vibratome sections of embryo showed in **v**–**x**. Note a moderate *GSN* expression within boundaries of dorsal root ganglia and in dermal cells (*short blue arrow*). There is a strong *GSN* expression in the cells surrounding pigmented retina (*black arrowhead*). *ao* aorta, *csp* caudal segmental plate, *dm* dermomyotome, *drg* dorsal root ganglia, *ev* eye vesicle, *hf* head fold, *ht* heart tube, *nl* neuroepithelial layer, *nt* neural tube, *ot* olfactory tract, *pa* pharyngeal arches, *sg* spinal ganglia, *sm* somatopleure, *sn* spinal nerve, *spm* splanchnic mesoderm, *so* somite, *sp* splanchnopleure, *va* visceral arches. The presence of staining within vesicles in the head region is due to the probe trapping. *Scale bars* *h, i, j, y* 1,000 μm, *h′*, *h″* 200 μm, *n*–*r* 500 μm

Having the antisense probe in hands recognizing the *GSN* transcript, we subsequently performed in situ hybridization experiments. In chicken embryos at HH-stage 8, we could observe relatively weak and homogenous *GSN* expression found mainly in the head fold and the caudal segmental plate (Fig. 2a). In embryos at HH-stages 10–11, a stronger *GSN* expression was observed in the eye vesicle, brain vesicles, the midbrain, the heart tube, the splanchnopleure, and the neural tube (Fig. 2b–d). We could not detect any *GSN* expression in the somites. In chicken embryos at HH-stage 18 (Fig. 2e–j), we observed distinct expression of *GSN* in cranial and dorsal root ganglia, in the atrioventricular canal, head mesenchyme, the eye cup, and in dermal cells underlying the epidermal ectoderm. Moreover, strong presence of *GSN* transcript was observed in mesenchymal cells surrounding the aorta (Fig. 2h′) and in the splanchnopleure, but not somatopleure in the posterior part of embryo (Fig. 2e, i, j). In older embryos at HH-stage 21, the expression pattern of *GSN* is still very distinct (Fig. 2k–r). High amount of *GSN* transcript were detected in cranial and dorsal root ganglia (Fig. 2k–r), the olfactory tract (Fig. 2l), the atrioventricular canal (Fig. 2l), the eye (Fig. 2k, l), and limbs mesenchyme (Fig. 2k). Analysis of a series of vibratome sections of chicken embryo shown in Fig. 2k revealed strong *GSN* expression not only in dorsal root ganglia but also in the sympathetic trunk (Fig. 2n–r). Interestingly, there is also a prominent presence of *GSN* transcript in the neuroepithelial and the mantle layers of the neural tube (Fig. 2q). In the chicken embryo at this developmental stage, we further observe *GSN* expression in mesenchymal cells surrounding the aorta and in dermal cells (Fig. 2q). In the chicken embryo at HH-stage 22 (Fig. 2s–u), the *GSN* expression pattern is slightly changed in comparison to stage HH21 chicken embryo. The signal in the cranial ganglia is not so distinct any more except for the trigeminal ganglion and a more prominent staining of pharyngeal arches is to be seen (Fig. 2t). Still, a strong *GSN* expression in dorsal root ganglia, the sympathetic trunk and limbs mesenchyme was observed. In chicken embryo at HH-stage 25 (Fig. 2v–x), we could observe the presence of *GSN* transcript especially in head and limbs mesenchyme, region of visceral arches, and dorsal root ganglia. The staining pattern of dorsal root ganglia in the proximal part (Fig. 2w) of the trunk was different from that in the posterior part (Fig. 2x). In vibratome sections of chicken embryo shown in Fig. 2v, we observed distinct *GSN* expression in dorsal root ganglia, dermal cells, and in the developing eye (Fig. 2y, z).

**Fig. 3:**
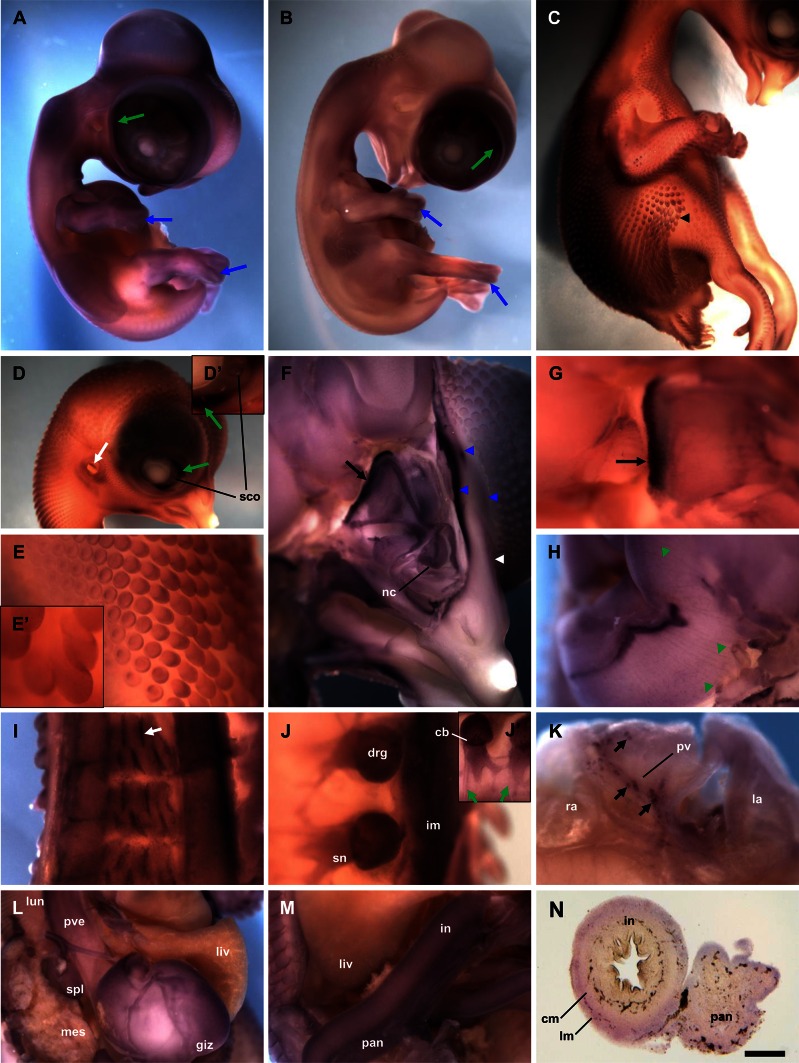
In situ hybridization experiments with the probe recognizing gelsolin transcript to whole mount chicken embryos at late stages. **a**–**m** Whole mount in situ hybridization. **n** Vibratome section. **a** Stage HH29 embryo. High *GSN* expression is observed in the eye (*green long arrow*), within the developing skin and in limbs mesenchyme (*long blue arrow*). The presence of staining within vesicles in the head region is due to the probe trapping. **b** Stage HH33 embryo. Note the high *GSN* expression level in the eye (*green long arrow*) and limbs mesenchyme (*long blue arrow*). **c**–**e** Stage HH36 embryo. There is a strong signal for gelsolin mRNA observed in nictitating membrane (*green long arrows*), at peripheries of scleral ossicles, in cochlea of ear (*white long arrow*) and feather buds (*black arrowhead* and **e**). **d′**, **e′** Higher magnifications of **d** and **e**, respectively. **f**–**n** Photos of cut longitudinal prior to the in situ hybridization embryo at HH36-stage. **f**–**h** High *GSN* expression is observed in the olfactory bulb (*long black arrow*), the nasal conchae, eye muscles (*blue arrowheads*), the developing comb (*white arrowhead*) and meninges. Note a line-patterned staining within meninges (*green arrowheads*). **i** Stained presumptive inter-innervation of neck skeletal muscles (*white short arrow*). **j** There is a high *GSN* expression in dorsal root ganglia, spinal nerves and presumably nerves within iliotrochantericus muscle. Note absence of signal in the more distal branches of the spinal nerves (*short green arrow*). **j′** Higher magnification of **j**. **k** Dorsal view of the atrial part of a heart, with strongly gelsolin mRNA-positive cells (*short black arrows*). **l**, **m** Note high *GSN* expression on the surface of proventriculus, gizzard, spleen, small intestine, and pancreas. **n** A vibratome section of intestine and pancreas shown in **m**. There is observed a strong signal for gelsolin mRNA within circular and longitudinal muscles of the intestine wall. *cm* circular muscle, *cb* cell bodies of neurons, *drg* dorsal root ganglia, *giz* gizzard, *im* iliotrochantericus muscle, *in* intestine, *la* left atrium, *liv* liver, *lm* longitudinal muscle, *lun* lung, *mes* mesonephros, *nc* nasal conchae, *pan* pancreas, *pv* pulmonary vein, *pve* proventriculus, *ra* right atrium, *sco* scleral ossicles, *spl* spleen, *sn* spinal nerve. *Scale bar* *n* 1,000 μm

In older chicken embryos at HH-stage 29, we observed a strong expression of *GSN* in the eye, limb bud mesenchyme, and the dermis (Fig. 3a). Later in the development at HH-stage 33, the presence of gelsolin mRNA was detected in the limb bud mesenchyme and eye (Fig. 3b). However, the overall signal was weaker when compared to younger stages (Fig. 3a). Next, we examined very detailed 10-day-old chicken embryo (HH-stage 36). We found strong *GSN* expression in the outer cell layer of the feather buds (Fig. 3c, e, e′), the cochlea of ear, the nictitating membrane and at peripheries of scleral ossicles (Fig. 3d, d′). To gain more information about *GSN* expression in organs and tissues of developing chicken embryo prior to in situ hybridization, we longitudinally cut chicken embryos into two parts. We found out that there are high amounts of *GSN* transcripts in the olfactory bulb, the nasal conchae, the developing comb, eye muscles (Fig. 3f, g), and meninges (Fig. 3h), where we can observe a line-patterned staining. We observed an interesting pattern of most probably inter-innervation of neck skeletal muscles (Fig. 3i) and back skeletal muscles, e.g., iliotrochantericus muscle (Fig. 3j). As in younger chicken embryos, we could observe a strong expression of *GSN* in the dorsal root ganglia, especially in cell bodies of neurons and spinal nerves crossing the dorsal root ganglia, but not in the more distal branches (rami) of the spinal nerves (Fig. 3j, j′). Presence of *GSN* transcript is manifested also in a set of cells localized in the atrial part of the heart (Fig. 3k), on the surface of proventriculus, gizzard, spleen, intestine, and pancreas. The signal for gelsolin apparently localized within smooth muscle layers of circular and longitudinal muscles of intestine. A characteristic staining pattern on the surface of gizzard suggests stained structures belong to enteric nervous system (Fig. 3l–n). In situ hybridization experiments with the sense probe served as negative controls and gave no specific stainings (Online Resource 1, Fig. S2).

### RT-PCR and western blotting experiments

**Fig. 4:**
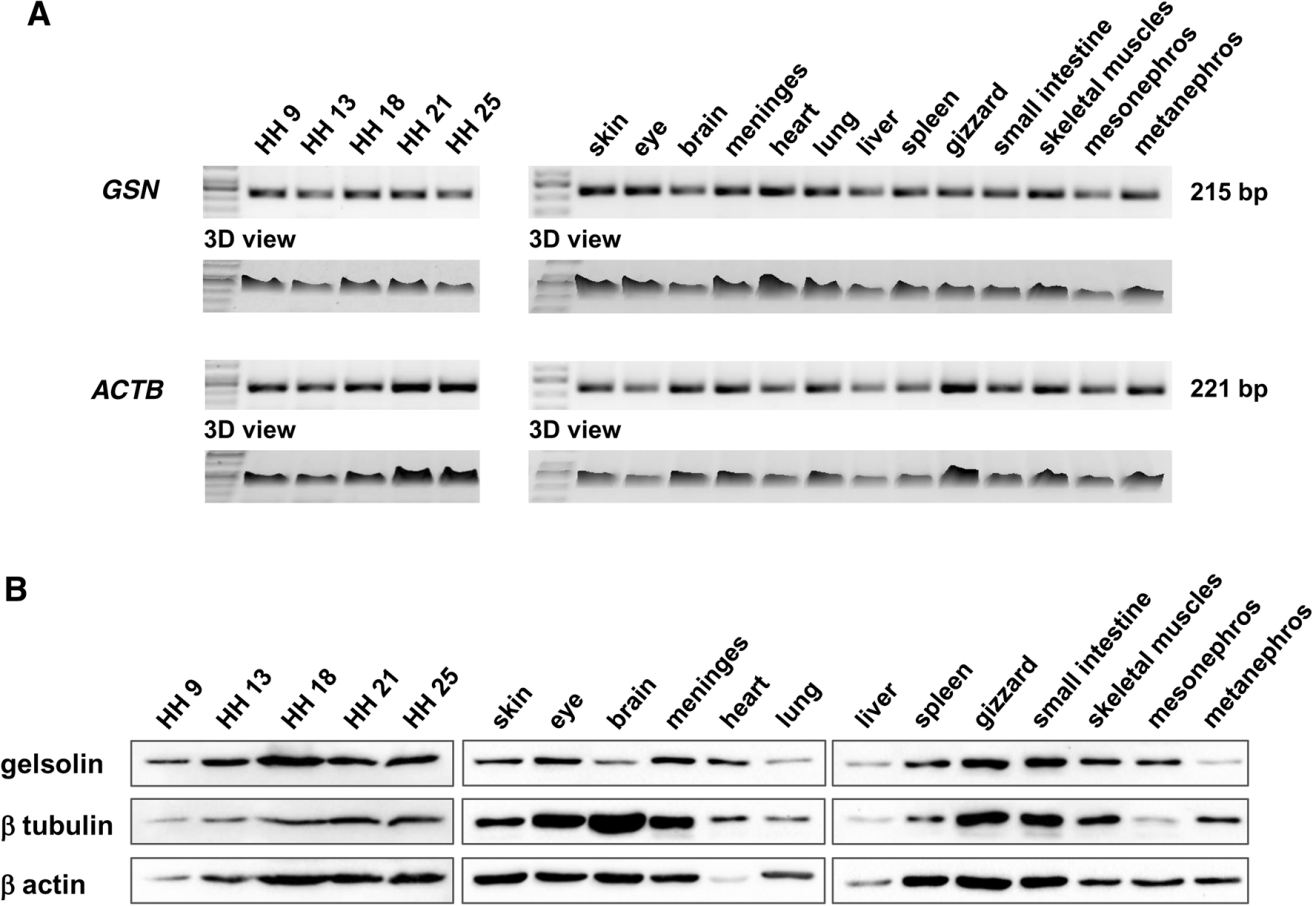
RT-PCR and Western blot analysis of gelsolin expression in chicken embryos and organs from chicken embryos at stage HH36. **a** Horizontal DNA electrophoresis of PCR products. As templates for PCR served cDNAs obtained from chicken embryos at different developmental stages (HH9-25) and tissues removed from chicken embryo at HH36 stage. PCRs were carried out using primers spanning exons recognizing *GSN* mRNA. *ACTB*, gene coding β actin served as a reference gene. Note different expression levels of *GSN* and *ACTB* genes in tested tissues. We observed highest amount of *GSN* product in skin, eye, meninges, heart, and lung. **b** Western blot analysis of protein extracts from chicken embryos at different developmental stages (HH9–25) and tissues removed from chicken embryo at HH36 stage for presence of gelsolin at protein level. High amount of gelsolin was in the eye, meninges, gizzard and small intestine; moderate amount of gelsolin was observed for skin, heart, spleen, skeletal muscles, and mesonephros and the lowest amount of gelsolin was detected in brain, lung, liver, and metanephros. As reference β tubulin and β actin were taken. 30 μg of protein was loaded on every line

To confirm results from in situ hybridization experiments, we next performed RT-PCR analysis to detect *GSN* mRNA. In Fig. 1b, the sites to which primers, which were used in this analysis, anneal are shown. We analyzed the obtained cDNA libraries from chicken embryos at different stages and several organs of chicken embryo at HH-stage 36. Gelsolin mRNA was present at all tested stages of chicken embryo development. Interestingly, we noted less PCR products for *GSN* transcript in chicken embryo at HH-stage 25 than in earlier stages, whereas *ACTB* PCR product amount rose proportionally to the developmental stage (Fig. 4a). In all tested organs of chicken embryo at HH-stage 36, the presence of *GSN* transcript was observed; however, its amount varied among tissues and we observed highest amount of the product in skin, eye, meninges, heart, and lung. Upon identification of antibodies recognizing avian gelsolin (Online Resource 1, Fig. S3A), we performed Western Blot analysis of lysates of younger chicken embryos and organs of chicken embryo at HH-stage 36 (Fig. 4b). Gelsolin was detected at protein level in chicken embryos at all tested stages. In organs of stage 36 chicken embryos, we noted different amounts of gelsolin. Gelsolin was abundantly found in the eye, meninges, gizzard, and small intestine. Moderate amount of gelsolin was observed in the skin, heart, spleen, skeletal muscles, and mesonephros. Low amounts of gelsolin were detected in brain, lung, liver, and metanephros. These results reflect RT-PCR analysis and the results obtained from in situ hybridization experiments. Additionally, we have probed the membranes with antibodies recognizing either β actin or β tubulin as reference proteins. The amount of β actin or β tubulin differed between tested tissues, although we loaded on every lane 30 μg of protein. It was shown for several times neither β actin nor β tubulin are suitable loading controls (Dittmer and Dittmer 2006; Eaton et al. 2013) and amounts of β actin or β tubulin are unequal between, e.g., different tissues or normal and pathologically changed samples of the same tissue origin (Eaton et al. 2013). In our case, there was for example trace amount of β actin in tissue extract of heart isolated from 10-day-old chicken embryo (Fig. 4b). Probably at this stage of development most of actin pool in chicken heart consists already of α cardiac actin. On the other hand, the highest amount of β tubulin was observed in the brain extract (Fig. 4b). Corresponding membranes to the WB analysis shown in Fig. 4b were subjected to a total protein analysis by staining with Ponceau S (Online Resource 1, Fig. S3B). Although it seems on some lanes (spleen or skeletal muscle) there was less protein loaded, it does not have to mean that in the initial protein mixture subjected to the WB analysis, there was an unequal amount of proteins. One has to keep in mind that we denatured the proteins by boiling them at 95 °C for 10 min. Membrane-associated proteins with a high degree of hydrophobicity aggregate under these conditions (Schägger 2006) and get stuck in the stacking gel resulting in their absence in the separating gel and finally on the membrane. Also, very small proteins (<10 kDa) can be problematic, when standard SDS-PAGE (Laemmli 1970) is applied (Schägger 2006). Here, we have used standard SDS-PAGE, because gelsolin, β actin, and β tubulin are well resolved under conditions of standard denaturing electrophoresis. One should consider our WB analysis as an overview of expression level of these three proteins in different tissues of developing chicken embryo at HH-stage 36 (10 days).

### Immunohistochemical stainings

**Fig. 5:**
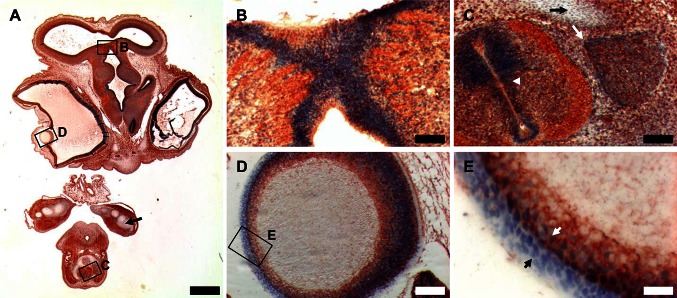
Immunohistochemical analysis of chicken embryo sections at stage HH33. Sections of chicken embryo were stained with rabbit monoclonal anti-gelsolin antibodies and counterstained with Mayer’s hematoxylin solution. **a** Frontal section of whole embryo. Note no signal in bone precursor tissues (*black arrow*). **b**–**e** Higher magnifications of section presented in **a**. **b** A strong signal was present in brain tissue. **c** There is high gelsolin amount in the ependymal layer of the neural tube (*white arrowhead*) and the dorsal root ganglion (*white arrow*). There is no signal for bone precursor tissue (*black arrow*). **d** There is high amount of gelsolin in the developing lens. **e** Higher magnification of the section presented in **d**. Note the basal cell layer of the lens epithelium is rich in gelsolin (*short white arrow*), whereas the apical cell layer of the lens epithelium is devoid of gelsolin (*short black arrow*). *Scale bar* *a* 1,000 μm, *b*–*d* 90 μm, *e* 25 μm

Having antibodies recognizing chicken gelsolin (Fig. 4 and Online Resource 1, Fig. S3A), we decided to analyze paraffin sections stained with these antibodies and counterstained with Mayer’s hematoxylin solution to gain more information about the distribution of gelsolin at protein level in embryos at stages HH33 and HH36. Analysis of stained frontal sections of chicken embryo at HH-stage 33 revealed the presence of high gelsolin amounts in the notochord/neural tube—especially in the ependymal layer of the neural tube, ganglia, dermomyotome, and under epidermis (Fig. 5a, c). Note the complete absence of signal for gelsolin in bone precursor tissue (Fig. 5c). In the brain, gelsolin was moderately present and relatively uniformly distributed (Fig. 5a, b). In the eye, we observed a strong signal for gelsolin within the iris and the lens. Intriguingly, the gelsolin rich cells were present in the basal layer of the lens epithelium, whereas the cells in the apical layer of the lens epithelium were devoid of gelsolin (Fig. 5d, e).

**Fig. 6:**
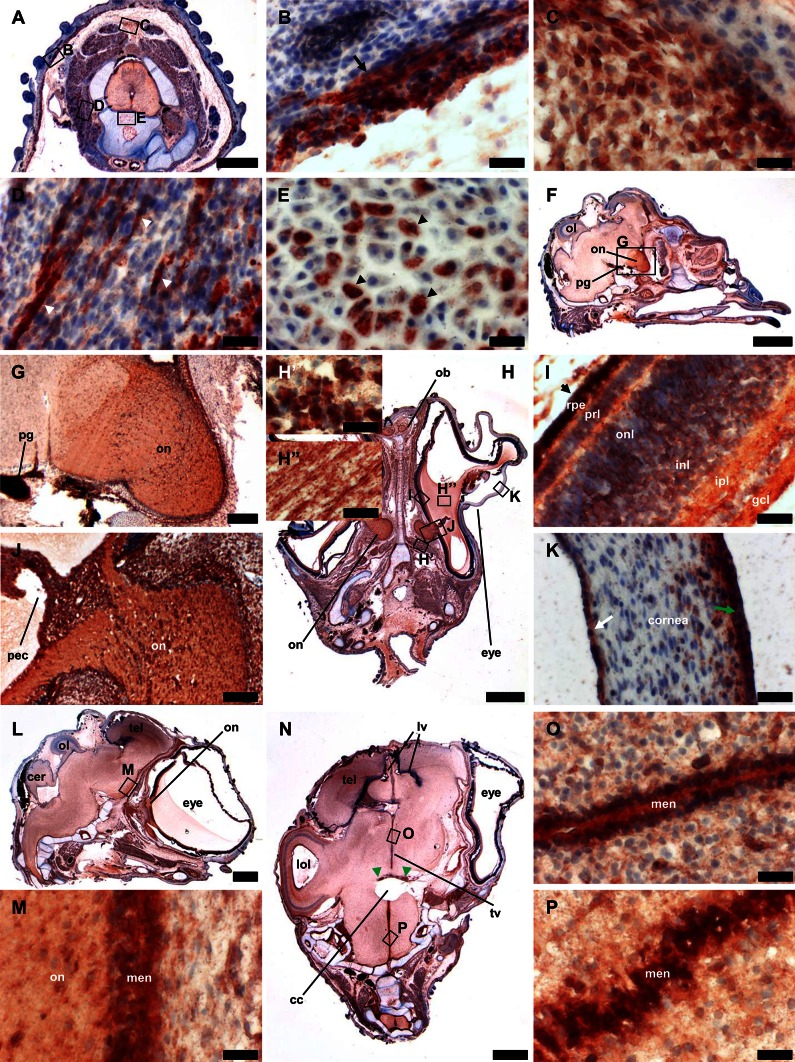
Immunohistochemical analysis of chicken embryo sections at stage HH36. Sections of chicken embryo were stained with rabbit monoclonal anti-gelsolin antibodies and counterstained with Mayer’s hematoxylin solution. **a** Transverse section of the neck. **b**–**d** Higher magnifications of section presented in **a**. **b** Note high gelsolin amount in the cells layer (*black arrow*) adjacent to the basement membrane. **c** There was a strong signal for gelsolin in most probably fibroblasts forming ligaments. **d** Within neck muscles there were strips of cells strongly positive for gelsolin (*white arrowheads*). **e** The cells within forming vertebra exhibited strong gelsolin presence (*black arrowheads*). **f** Sagittal section of the head. **g** Higher magnification of section presented in **f**. Note higher amount of gelsolin in the optic nerve and presumably the pituitary gland than in the rest of cerebral hemisphere. **h** Frontal section of the head. **h′**–**k** Higher magnifications of the section presented in **h**. **h′** Note a strong signal for gelsolin in muscle fibers of the external eye muscle. **h″** There was relatively high amount of gelsolin in the vitreous chamber of the eye. **i** In all layers of the eye retina, we noted gelsolin presence including the retinal pigment epithelium (*short black arrow*). **j** Note high amount of gelsolin in the optic nerve and the pecten. **k** In the epithelial layer (*green arrow*) and in the endothelial layer (*white arrow*) of the cornea, there was detected a very high gelsolin level. **l** Sagittal section of the head. **m** Higher magnification of section presented in **l**. The cells building meninges exhibit high amount of gelsolin. Note high amount of gelsolin in telencephalon. **n** Frontal section of head. Again high amount of gelsolin was observed in the telencephalon, meninges within ventricles and in branches of oculomotor nerve (*green arrowheads*). **o**, **p** Higher magnifications of the section presented in **n**. High amount of gelsolin was observed in meninges between two hemispheres. *cc* central canal, *cer* cerebellum, *gcl* ganglion cell layer, *ipl* inner plexiform layer, *inl* inner nuclear layer, *lol* lumen of optic lobe, *lv* lateral ventricle, *men* meninges, *ob* olfactory bulb, *on* optic nerve, *onl* outer nuclear layer, *pec* pecten, *pg* pituitary gland, *prl* photoreceptor layer, *rpe* retinal pigment epithelium, *tel* telencephalon, *tv* third ventricle. *Scale bar* *a* 500 μm, *b*–*e*, *h*′–*h*″, *i*, *k*, *m*, *o*–*p* 25 μm, *f* 2,000 μm, *g* 200 μm, *h, l, n* 1,000 μm, *j* 60 μm

Because the results of in situ hybridization experiments suggested that *GSN* is especially expressed in some tissues of ectodermal origin including neural crest-derived cells, we decided to get a closer look at the heads of chicken embryos at HH-stage 36. We observed a strong signal for gelsolin in several tissue types (Fig. 6 and Online Resource 1, Fig. S4). Interestingly, while analyzing transverse sections of neck, we noted a very high amount of gelsolin in cells adjacent to the basement membrane (Fig. 6a, b), in strips of cells within neck muscles (Fig. 6a, d), most probably in fibroblasts, which form ligaments (Fig. 6c) and in the cells within the vertebral cartilage (Fig. 6e). Analysis of head sagittal sections revealed the presence of gelsolin within the developing brain with significantly stronger presence of gelsolin in the optic nerve and presumably the pituitary gland (Fig. 6f, g, l, m), meninges and the telencephalon (Fig. 6l, m). High amounts of gelsolin in meninges, the telencephalon and branches of oculomotor nerve were also detected in analyzed frontal sections of chicken heads (Fig. 6n, p). Frontal section of embryo heads (Fig. 6h) showed high amounts of gelsolin in the optic nerve and the pecten (Fig. 6j) and in the cells within muscle fibers of ventral oblique muscle of the eye (Fig. 6h′). High amounts of gelsolin were observed in tissues of the eye (Fig. 6h and Online Resource, Fig. S4F). Strong signal was noted in the vitreous chamber of eye (Fig. 6h″), the retina, the choroidea, the sclera (Fig. 6i and Online Resource 1, Fig. S4F), the iris, the conjunctiva (data not shown) and finally in the epithelial and endothelial layers of cornea, whereas the stroma of the cornea was gelsolin-free (Fig. 6k). Moreover, high amount of gelsolin was observed in brain pericytes (Online Resource 1, Fig. S4D), muscle-connective tissue in the walls of head vessels (Online Resource 1, Fig. S4E), nerves within head (Fig. 6n and Online Resource 1, Fig. S4I), olfactory bulb (Fig. 6h) and the olfactory epithelium of the nasal conchae (Online Resource 1, Fig. S4G, H). Interestingly, we noted different patterns of staining within muscles, i.e., all muscle fibers within the head were gelsolin-positive (Online Resource 1, Fig. S4B, C), whereas within neck musculature the staining showed a strip-like pattern (Fig. 6d and Online Resource 1, Fig. S4A). In some feather buds, we noted the absence of gelsolin at protein level (Fig. 6a), whereas in feather buds localized, e.g., on the head, we observed a strong gelsolin presence (Online Resource 1, Fig. S4J, K).

As a summary of *GSN* expression in chicken embryos between developmental days 1 and 10, we assembled the obtained data in a table (Table 2).

**Table 2:** Expression of *GSN* in tissues of analyzed embryos at HH-stages 8–36 (E 1–10)

HH-stage (E)	Tissue
HH-stage 8 (E 1)	Head fold^a^, caudal segmental plate^a^, whole embryo analysis^b,c^
HH-stage 10–11 (E 1.5–2)	Eye vesicle^a^, brain vesicles^a^, midbrain^a^, heart tube^a^, splanchnic mesoderm^a^, neural tube^a^, whole embryo analysis^b,c^
HH-stage 18 (E 3)	Head mesenchyme^a^, eye cup^a^, atrioventricular canal^a^, cranial ganglia^a^, dorsal root ganglia^a^, splanchnopleure^a^, mesenchymal cells surrounding aorta^a^, atrioventricular canal^a^, dermal cells underlying the epidermal ectoderm^a^, whole embryo analysis^b,c^
HH-stage 21–22 (E 3.5)	Eye cup^a^, olfactory tract^a^, cranial ganglia^a^, dorsal root ganglia^a^, spinal ganglia^a^, sympathetic trunk^a^, spinal nerve^a^, neuroepithelial layer of neural tube^a^, mantle layer of neural tube^a^, atrioventricular canal^a^, limbs mesenchyme^a^, mesenchymal cells surrounding aorta^a^, dermal cells underlying the epidermal ectoderm^a^, pharyngeal arches^a^, whole embryo analysis^b,c^
HH-stage 25 (E 5)	Eye^a^, head mesenchyme^a^, limbs mesenchyme^a^, visceral arches^a^, dorsal root ganglia^a^, dermal cells^a^, cells surrounding pigmented retina^a^, whole embryo analysis^b,c^
HH-stage 29 (E 6–6.5)	Eye^a^, within the developing skin^a^, limbs mesenchyme^a^
HH-stage 33 (E 8)	Eye structures^a,d^, limbs mesenchyme^a^, brain^d^, ependymal layer of neural tube^d^, dorsal root ganglia^d^
HH-stage 36 (E 10)	Eye structures^a,b,c,d^, cochlea of ear^a^, olfactory bulb^a,d^, nasal conchae^a,d^, eye muscles^a,d^, developing comb^a^, meninges^a,b,c,d^, brain^b,c^, nerves within head^d^, pituitary gland ^d^, presumptive inter-innervation of skeletal muscles^a,d^, skeletal muscles^b,c,d^, dorsal root ganglia^a,d^, spinal nerves^a^, cells in the atrial part of the heart^a^, heart^b,c^, lung^b,c^, liver^b,c^, proventriculus^a^, gizzard^a,b,c^, spleen^a,b,c^, small intestine^a,b,c^, pancreas^a^, circular and longitudinal muscles of the intestine wall^a^, skin^b,c,d^, feather buds^a,d^, mesonephros^b,c^, metanephros^b,c^, population of cells within forming vertebra^d^, pericytes in the head^d^

^a^In situ hybridization

^b^RT-PCR

^c^WB

^d^Immunohistochemistry

### Gelsolin in chicken brain development

**Fig. 7:**
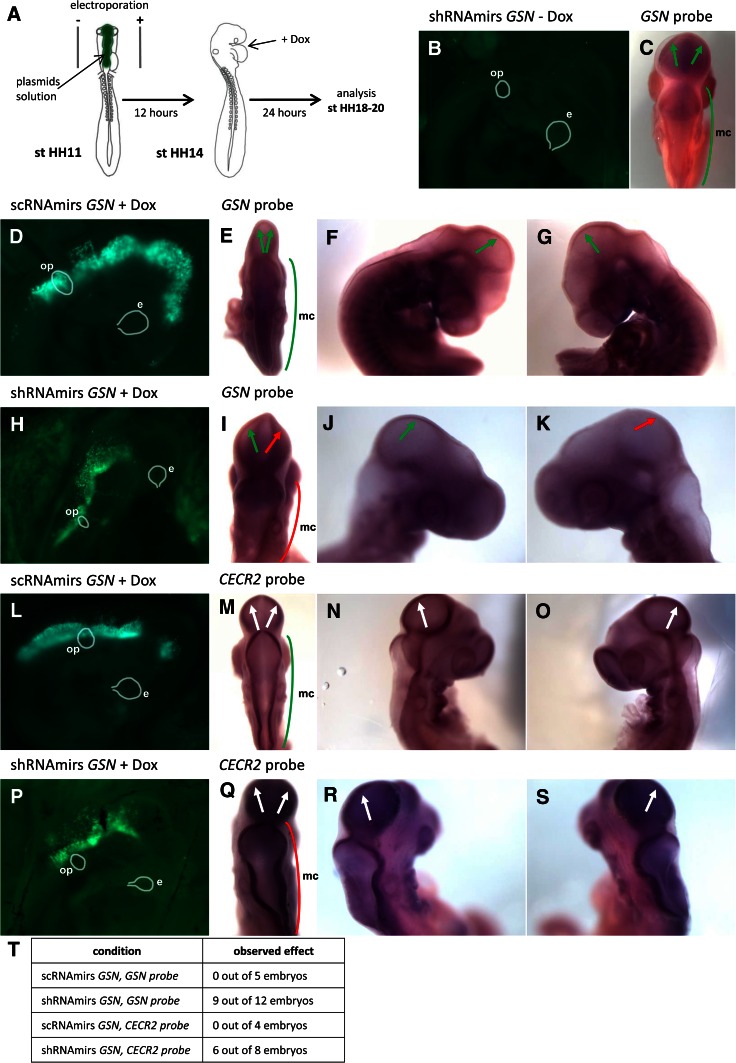
Analysis of electroporated chicken embryos expressing EGFP, scRNAmirs, or shRNAmirs targeting gelsolin mRNA. **a** A diagram showing HH-stages, at which embryos were electroporated, treated with doxocycyline and harvested. **b**, **d**, **h**, **l**, **p** Photos of EGFP fluorescence taken in ovo. **c**, **e**–**g**, **i**–**k**, **m**–**o**, **q**–**s** Whole mount in situ hybridization. **c**, **e**–**g**, **m**–**o** *GSN* probe was used. **i**–**k**, **q**–**s** *CECR2* probe was used. **b**–**c** Control experiment showing an electroporated embryo without triggered expression of shRNAmirs targeting gelsolin. Note no fluorescence signal of EGFP, no changes within right part of mesencephalon (*green line*) and unaltered *GSN* expression level in both parts of optic tectum (*green arrows*). **d**–**g** Embryo expressing scRNAmirs and EGFP (**d**). There are no changes within right part of mesencephalon (**e**, *green line*) and no decrease in *GSN* expression within optic tectum (*green arrows*). **h**–**k** Electroporated embryo expressing shRNAmirs and EGFP (**h**). Note shortened right part of mesencephalon (**i**, *red line*) and diminished *GSN* expression within right part of optic tectum (*red arrow*), but not the left one (*green arrow*). The changes in the *GSN* expression level in the right part of the optic tectum were observed, when the embryo was photographed from the left, non-electroporated side of the embryo (**k**). In contrast, no changes in *GSN* expression level in non-electroporated, left side of the embryo were observed from the right, electroporated side of the embryo (**j**). **l**–**o** Embryo expressing scRNAmirs and EGFP (**l**) showed no changes in the right part of the mesencephalon (**m**, *green line*) and no decrease in *CECR2* expression level (*white arrows*). **p**–**s** An embryo expressing shRNAmirs and EGFP (**p**). Note the changed structure of the right part of the mesencephalon (**q**, *red line*), but there are no alterations in expression level of *CECR2* (*white arrows*). **t** Statistical summary of abundance of observed effect, i.e., shortened right part of mesencephalon. *e* eye, *mc* mesencephalon, *op* otic placode/vesicle

Because we have noted high *GSN* expression level especially in the head regions, we asked ourselves, if gelsolin is important for chicken brain development. For this purpose, we have generated plasmids coding for shRNAmirs targeting gelsolin mRNA. We have electroporated chicken embryos into brain vesicles at HH-stages 11–12 with plasmids solution. These stages are the most suitable for our approach, because the neural tube is already closed and the head of the embryo is still unturned. From HH-stage 14, it is impossible to electroporate the brain vesicles in ovo because the head is turned completely to the right, what makes impossible to perform electroporation of one side of the brain vesicles. To trigger expression of shRNAmirs and EGFP 12 h later, we have injected doxycycline between the embryo and yolk. After 24 h, we photographed the embryos in ovo and harvested them. The embryos developed to HH-stages 18–20. We tried to incubate the embryos for longer times, but we were not successful. Two types of control experiments were carried out: (1) we did not induce expression of shRNAmirs and EGFP by applying doxycycline solution (Fig. 7b, c) or (2) we used plasmids coding scRNAmirs (Fig. 7d–g, l–o). Following harvesting of the embryos, in situ hybridization experiments were performed. We used the probes recognizing mRNAs coding gelsolin and Cecr2. Expression of *CECR2* was observed in the developing chicken brain (Chen et al. 2010), that is why we have used it as a brain marker to track potential morphological changes within the brains of manipulated embryos. We noted in embryos expressing a mixture of shRNAmirs targeting gelsolin mRNA lowered expression of the gene encoding gelsolin (Fig. 7i–k), but not of *CECR2* (Fig. 7q–s). We observed a striking effect of *GSN* expression silencing concerning development of mesencephalon, i.e., the electroporated part of the mesencephalon seemed to be underdeveloped resulting in a shortened right part of the mesencephalon in comparison to the non-electroporated, left part of the mesencephalon (Fig. 7i–k, q–s, t). We did not note the described effect in control experiments (Fig. 7c, e–g, m–o, t).

## Discussion

There is very little data concerning gelsolin in chicken development. Heller et al. (1998) identified mRNAs of a group of proteins highly abundant in the inner ear of the chicken. One of “tissue-specific” mRNAs found in the homogeneous cells was mRNA for a protein named by the authors homogenin. The researchers, applying Northern blot analysis, found out homogenin’s transcript was present in high amount also in the heart and the skin and in lower amount in muscles, the cerebellum, the forebrain, and the eye. However, the comparison of amino acid sequences of chicken gelsolin and homogenin revealed homogenin is 100 % identical to chicken gelsolin (Online Resource 1, Fig. S5). In 2013, Korte and colleagues published proteomic data confirming for the first time the presence of gelsolin at protein level in chicken. Their analysis suggests gelsolin is potentially important for development of chicken bursa of Fabricius. Interestingly, they showed the highest amount of gelsolin at embryonic day 18 suggesting gelsolin could be of significant relevance for the development of bursa of Fabricius during bursal stage. Lower amounts of gelsolin were noted in the prebursal and postbursal stages.

In this paper, we show for the first time the distribution of gelsolin not only at transcript but also at protein level in the developing chicken embryo from embryonic day 1 (E 1) to E 10. At the early stages of the embryonic development, i.e., HH10–11, we observed the presence of gelsolin mRNA in the eye and brain vesicles, the midbrain, the heart tube, the splanchnopleure and the neural tube, but not in the somites. Later on, at the HH18 stage *GSN* was expressed apparently in all embryonic tissues. However, an enhanced expression was noted in the eye, cranial, and dorsal root ganglia, the atrioventricular canal, dermal cells, and the splanchnopleure. In older, at the HH21 stage embryo *GSN* was highly expressed in all cranial and dorsal root ganglia and the sympathetic trunk suggesting gelsolin could be important for the development of the peripheral nervous system (PNS). We noted strong *GSN* expression also in the olfactory tract and the developing eye. In older embryos (HH36), a very strong presence of gelsolin mRNA was observed in the cochlea, the olfactory bulb, the eye, eye muscles, organs (proventriculus, gizzard, spleen, intestine, and pancreas), presumably skeletal muscle innervations, meninges, the heart, feather buds, and within the developing dermis. Our observations concerning gelsolin mRNA distribution were corroborated by RT-PCR, Western Blot and immunohistochemical analyses. We decided to focus on the head, because the results from in situ hybridization experiments suggested *GSN* is especially highly expressed in several tissues of ectodermal origin including neural crest (NC) derivatives, which give origin to many tissues within the head (Le Douarin et al. 2004; Dupin and Sommer 2012). We observed the presence of gelsolin at mRNA and protein level only in a part of tissues of ectodermal origin: the olfactory epithelium, the olfactory bulb and tract, the lens, elements of the optic cup, the optic nerve, branches of the oculomotor nerve, telencephalon, and presumptive the pituitary gland. Interestingly, we found out that *GSN* was expressed in all analyzed by us NC-derived cell lineages, e.g., in the endothelium of the cornea, the cochlea, pericytes of the brain; meninges, walls of blood vessels, developing dermis and muscles within the head. According to the literature, NC derivatives are found, e.g., in heart (Deal et al. 2006; Levin et al. 2009; Simon et al. 2012), eye structures (Simon et al. 2012; Dupin and Sommer 2012; Swamynathan 2013), cochlea (Simon et al. 2012), skin and feather buds (melanocytes), Schwann cells of gut, stomach and sciatic nerve (Hari et al. 2012; Simon et al. 2012; Dupin and Sommer 2012), meninges, pericytes of the brain (Le Douarin et al. 2004; Simon et al. 2012; Dupin and Sommer 2012), muscle-connective tissue walls of blood vessels within the head (Le Douarin et al. 2004) and ensheathing cells within the olfactory nerve (Dupin and Sommer 2012).

Although, in the brain of older chicken embryos (E 10), we observed a higher gelsolin expression only in the brain pericytes and telencephalon, but not in other brain cell types, we decided to test if gelsolin is important for chicken brain development. We silenced *GSN* expression by electroporation of the brain vesicles of chicken embryo at HH-stages 11–12 with plasmids coding shRNAmirs targeting gelsolin mRNA. Because of technical limitations, we could analyze only young embryos. However, we were able to observe a morphological effect of *GSN* expression silencing. In most of the analyzed embryos expressing shRNAmirs targeting gelsolin mRNA, the mesencephalon was shorter in comparison to control embryos. This suggests gelsolin is crucial for proper development of the chicken brain.

The *GSN* expression pattern presented on vibratome sections of chicken embryos at HH-stage 21 resembles that one described by Adameyko et al. (2009). The authors showed melanocytes in birds and mammals originate in two ways, either in dorso-lateral or ventral pathway. In the former case, melanoblasts delaminate directly from the roof plate of neural tube and in the latter case melanocytes are derived from Schwann cell precursors (SCPs) surrounding peripheral nerves. The ventral pathway gives rise to melanocytes found in the ventral part of the body and in the limbs both within epidermis as well as extracutaneous. The exact mechanism deciding if SCPs will become Schwann cells or melanocytes is still to be elucidated. The *GSN* expression pattern observed by us especially around developmental stage HH21, where we observed a strong signal for gelsolin mRNA in the dorsal root ganglia, spinal nerves, and its branches, resembles expression pattern of *SOX2* and *SOX10* genes, which products are involved in the peripheral nerves and melanocyte development (Adameyko et al. 2012). Moreover, we noted enhanced *GSN* expression within the developing dermis corroborating our notion that *GSN* is expressed most probably also in melanoblasts migrating along the dorso-lateral pathway to their destinations. Altogether it supports again our thesis that *GSN* expression is enhanced in the NC-derived cells.

Tanaka and Sobue (1994) observed in rats the presence of gelsolin in Schwann cells of PNS and in some structures of the CNS, although with different abundancy. Highest amount of gelsolin was observed in basal regions of the brain. A detailed analysis revealed gelsolin was localized in the myelin sheath of oligodendrocytes and Schwann cells in the CNS and PNS, respectively. *GSN* expression was changing in time and the highest amount of gelsolin at protein levels in the brain were observed 3–4 weeks after birth, upon this period, the gelsolin amount was decreased; however, even in 6-month-old rats, gelsolin was detectable in oligodendrocytes. Gelsolin was observed neither in neurons nor in other types of glial cells. We noted also that most probably innervation of proventriculus, gizzard, spleen, intestine, and pancreas was positive for the presence of gelsolin mRNA, which again is in accordance with the literature saying *GSN* expression is observed in Schwann cells in the peripheral nerves (Tanaka and Sobue 1994). Although high gelsolin amounts were found in the myelin sheath producing cells, it seems that this ABP is not essential for the remyelination process after nerve injury of peripheral nerves in mouse (Gonçalves et al. 2010). Our immunohistochemical analysis of the developing chicken brain revealed higher *GSN* expression levels within telencephalon and the presumptive pituitary gland, whereas gelsolin-positive pericytes were found in the whole brain. We did not observe other cells, e.g., oligodendrocytes in the brain of E10 chicken embryo, which would be strongly gelsolin-positive. Although oligodendrocytes are not of neural crest origin, it is surprising that in the adult, but not embryonic mouse oligodendrocytes, *SOX10* expression reporter cells were noted (Simon et al. 2012). It could be possible that first upon initiation of *SOX10* expression in the brain of hatchlings, *GSN* expression in oligodendrocytes would begin. Due to that it could be possible that *GSN* expression is controlled by a signaling pathway involving Sox10 in both cell types: oligodendrocytes and Schwann cells. Interestingly, Sox10 is also crucial for myelin sheath maintenance in adult mice (Bremer et al. 2011). Tanaka and Sobue (1994) tested rats after birth, but there were no experiments performed on embryos at different developmental stages. That is why it is not known, if gelsolin is present in the rat embryonic oligodendrocyte lineage. Intriguing is also the fact that in the adult mouse cranial pericytes *SOX10* is not expressed, whereas it is in the embryonic pericytes (Simon et al. 2012). Up to date, there are no studies concerning the correlation of *GSN* expression with *SOX10* expression. *SOX10* expressing cells were found in mouse embryonic cochlea, cranial, and dorsal root ganglia, skin, gut, stomach, eye, hair follicles, melanocytes, peripheral nerves, and adrenal gland (Simon et al. 2012). We observed enhanced *GSN* expression in the same tissues of developing chicken embryo except for adrenal gland, which was not analyzed by us. The presumption that Sox10 could influence *GSN* expression is additionally supported by the fact that in patients with schizophrenia the *SOX10* methylation level was inversely correlated with the expression level of several “oligodendrocyte” genes including *GSN* (Iwamoto et al. 2005). We noted decreased *GSN* expression and gelsolin amount in several melanoma cell lines in comparison to normal melanocytes (our unpublished data), corroborating the thesis that gelsolin is a tumor suppressor (Li et al. 2012). Even though gelsolin amounts were decreased in human melanoma cells, they expressed *GSN* at a relatively higher level than other tumor types (our unpublished data) (Litwin et al. 2012). Interestingly, it was reported *SOX10* is expressed in melanoma cells (Deal et al. 2006; Shakhova et al. 2012), where it is apparently involved in formation and progression of melanoma (Shakhova et al. 2012). Intriguingly, in situ hybridization experiments revealed a population of gelsolin-positive cells in the upper part of the chicken heart at E10 (HH36). NC-derived cells were found in the corresponding region in the mouse heart (Deal et al. 2006; Levin et al. 2009). These cells apart from Sox10- were Dct-, Mitf-, and Tyr-positive suggesting they are melanocyte-like. They also expressed adrenergic and muscarinic receptors. This together with the fact these cells most probably directly contact both autonomic nerves and cardiomyocytes suggests these melanocyte-like cells could be arrhythmogenic triggers (Levin et al. 2009). It is of course possible that other signaling pathways play a role in neural crest cells fate regulation (Milet and Monsoro-Burq 2012) and thus upstream of Sox10 could regulate *GSN* expression during embryonic development and formation of melanocytes and melanoma in adult organisms.

Although gelsolin is considered as a tumor suppressor in many types of malignancies, e.g., breast, bladder, colon, gastric, kidney, lung, oral, ovarian, pancreatic, and prostate cancers (reviewed by Li with colleagues (Li et al. 2012)), there is very little known about gelsolin in CNS and PNS tumors. Ohnishi et al. (2009) reported gelsolin could be a potential marker of astrocytomas, since gelsolin amount was inversely correlated with tumor aggressiveness as stated by analyzing cerebrospinal fluid of patients with astrocytoma. In this study, we observed strongly gelsolin-positive pituitary gland. In this context, it is interesting to mention that in a reported case of pituicytoma, a very rare glioma of the pituitary gland of unknown cell origin, there were observed gelsolin amyloid deposits. However, there was not found any mutation in *GSN* (Ida et al. 2013).

More data about gelsolin in NC derivatives comes from studies concerning a very rare autosomal dominant disease: gelsolin amyloidosis known also as the familial amyloidosis of Finnish type (FAF). Single point mutation in *GSN* leads to generation of a protein, where in the position 187 instead of aspartic acid either asparagine or tyrosine residue is present. Interestingly, this substitution is amyloidogenic only in case of plasma gelsolin (isoform a). Only this isoform prior to secretion goes through endoplasmic reticulum and trans-Golgi network, where plasma gelsolin^D187N/Y^ is cleaved by furin (α-gelsolinase). Next, truncated plasma gelsolin^D187N/Y^ is cleaved by metalloproteinases, e.g., MT1-MMP in extracellular space causing occurrence of amyloidogenic fragments. First manifestations of gelsolin amyloidosis are lattice dystrophy of the cornea and amyloid depositions found in eye elements: the sclera, the conjunctiva, nerves, and vessels. Next, cranial and peripheral nerves are affected, what leads to neuropathy. The skin is also affected by accumulation of amyloid deposits adjacent to basement membrane resulting in cutis laxa and in later phase of disease proteinuria and amyloid deposits in blood vessels occur (Solomon et al. 2012). Interestingly, in patients with FAF heart conduction abnormalities were observed such as atrio-ventricular block (Chastan et al. 2006) or arrhythmias (Stewart et al. 2000). Paunio (1998), applying in vitro techniques, showed that in contrast to earlier assumptions, not muscles, but cells of neural origin are most probably responsible for the secretion of truncated plasma gelsolin^D187N/Y^. Surprisingly, according to the authors, Schwann cells process only up to 7 % of mutated form of plasma gelsolin, whereas cells of neuronal origin cleave 60–90 % of plasma gelsolin^D187N/Y^ into amyloid fragments. However, keeping in mind FAF is manifested first in the third decade of life, it is plausible that low level of processing of mutated plasma gelsolin by Schwann cells could be possible. Nevertheless, it is also possible that other neural crest derivatives such as sensory neurons could also secrete plasma gelsolin^D187N/Y^, since patients with FAF had reduced sensation (Kiuru 1998). The character of gradually occurring symptoms of FAF supports our observations of enhanced *GSN* expression in some cell lineages of ectodermal origin and in all NC derivatives in the developing chicken.

## Future goals

Although we observed enhanced *GSN* expression in NC derivatives, the biological significance of this phenomenon is unsolved and raises important questions. NC cells are highly motile and undergo EMT (Kuriyama and Mayor 2008). Together with the notion that gelsolin is a tumor suppressor, it suggests gelsolin’s role in NC cells development can have other implications, than simply regulating the actin cytoskeleton and thus influencing cell motility. Another issue is that there were observed different gelsolin expression patterns in head muscles and skeletal muscles of, e.g., the neck. Western blot analysis performed by Kwiatkowski et al. (1988) revealed high *GSN* expression level in human and rat skeletal muscles. Thus, further studies are needed to identify gelsolin-positive cell types within skeletal muscles, especially in correlation to developmental stage and phase of life. Stunning is also the high *GSN* expression level in the splanchnopleure. Because gelsolin is indispensable for embryonic development in lower vertebrates playing most probably a signaling function (Kanungo et al. 2003) and gelsolin downregulation is of high importance during tumor transformation in humans (Li et al. 2012), more effort should be put into the precise understanding of the role of gelsolin in development and cell motility. Among future goals there should also be the functional analysis of gelsolin in NC cells and in myelin producing cells in CNS and PNS. It should furthermore be elucidated by functional studies, if gelsolin is crucial for the chicken embryonic development like in zebrafish. Another issue to be elucidated is the estimation if *GSN* expression is correlated with Sox10 involving pathways and if gelsolin is present in oligodendrocytes of the adult chicken brain. Moreover, it would be of considerable importance to check, if in chicken more than one gelsolin isoform is present. Identification of antibodies recognizing chicken gelsolin allows for more detailed analysis of gelsolin presence in organs from older chicken embryos, hatchlings, and adult animals, which could bring more valuable data for comparative studies and thus help to understand the role of gelsolin in NC derivatives and some cell lineages of ectodermal origin such as oligodendrocytes or cells becoming constituents of the optic and olfactory systems.

## Electronic supplementary material

Below is the link to the electronic supplementary material.

Supplementary material 1 (PDF 2529 kb)

## Abbreviations

ABPs: Actin-binding proteins
CNS: Central nervous system
Dox: Doxycycline
E: Embryonic day
EGFP: Enhanced fluorescent protein
*Gd*: *Gallus domesticus*
*GSN*: Gene coding gelsolin
HH-stage: Chicken developmental stage according to Hamburger and Hamilton
*Hs*: Homo Sapiens
NC: Neural crest
PNS: Peripheral nervous system
scRNAmir: microRNA-adapted scrambled RNA
shRNAmir: microRNA-adapted short hairpin RNA
