# Supplementary material for: Analysis of gelsolin expression pattern in developing chicken embryo reveals high GSN expression level in tissues of neural crest origin
Source: Brain Struct Funct. 2014 Oct 29;221(1):515–34. doi: 10.1007/s00429-014-0923-5 (PMC4720725; doi:10.1007/s00429-014-0923-5)
Supplement: Supplementary file 1 — Supplementary material 1 (PDF 2529 kb) [file 429_2014_923_MOESM1_ESM.pdf]

Brain Structure and Function

Analysis of gelsolin expression pattern in developing chicken embryo reveals high *GSN* expression level in tissues of neural crest origin

Antonina J. Mazur[1], Gabriela Morosan-Puopolo[2], Aleksandra Makowiecka[1], Maria Malicka-Błaszkiewicz[1], Dorota Nowak[1] and Beate Brand-Saberi[2]

[1]Department of Cell Pathology, Faculty of Biotechnology, University of Wroclaw, Poland

[2]Department of Anatomy and Molecular Embryology, Ruhr University, Bochum, Germany

Corresponding author:

Dr Antonina Joanna Mazur
ajmazur@ibmb.uni.wroc.pl
Department of Cell Pathology
Faculty of Biotechnology
University of Wroclaw
ul. Joliot-Curie 14a
50-383 Wrocław
tel. + 48 71 37 56 206

Materials and Methods

Bioinformatics analysis

Amino acid sequences alignments were performed using MAFFT, the multiple alignment program for amino acid or nucleotide sequences, version 7 (Katoh et al. 2005; Katoh and Standley 2013). Following amino acid sequences were analyzed: human gelsolin isoforms a (NP_000168.1, Pubmed), chicken gelsolin (Pubmed: NP_990265.1) and chicken homogenin (Pubmed: AAC62928.1). The structure of inactive gelsolin having following PDB accession code: 3FFN (Nag et al. 2009) was visualized with Swiss PDB Viewer.

Cloning

Dermis of chicken embryo at stage HH36 (E10) was dissected, followed by rinsing in ice-cold PBS, next homogenized by cutting and frozen at -80°C. After tissue thawing total RNA was isolated with the help of NucleoSpin® RNA II Kit (Macherey-Nagel). 1 µg of RNA was reverse transcribed using High Capacity cDNA Reverse Transcription Kit (Applied Biosystems) following the manufacturer's instructions. In order to sequence the whole DNA coding chicken gelsolin three PCRs were conducted to obtain three DNA fragments, which were subsequently cloned into p3xHA plasmid (Müller et al. 2012). Following primers were used: fragment A forward, 5'-ATGGGAAAGCAGGGCTTTGG-3', reverse, 5'-ATGGGAAAGCAGGGCTTTGG-3'; fragment B forward, 5'-CTGGTTAGGAGATGAAAGCTC-3', reverse, 5'-CTGGTCCTTGTCCCTCCAGTTC-3'; fragment C forward, 5'-CTGAGAGTGGTGAAACAC -3', reverse, 5'-TTAGACATCCACATCTGC-3'. It was impossible to obtain in a single PCR a PCR fragment coding for the whole gelsolin moiety. Fragment A forward primer annealed there strongly to the gelsolin cDNA ca. 830 bp downstream of ATG codon. Phusion™ High-Fidelity DNA Polymerase (Thermo Scientific) was used to perform PCRs. This polymerase's error rate is 6-fold lower than that of *Pyrococcus furiosus* (*Pfu)* polymerase. Three obtained plasmids were sequenced and based on the results a DNA sequence coding the whole chicken gelsolin was assembled.

Isolation of cellular and tissue extracts

For Western blotting analysis human melanoma A375 cells and dermis from chicken embryos at HH-stages 26 and 36 were lysed on ice with cytoskeletal-bound protein extraction buffer (10 mM Tris-HCl, pH 7.4, 100 mM NaCl, 1 mM EDTA, 1 mM EGTA, 1 mM NaF, 20 mM $Na_4P_2O_7$, 2 mM Na_3VO_4, 1% Triton X-100, 10% glycerol, 0.1% SDS, 0.5% sodium

deoxycholate) supplemented with proteases inhibitors cocktail (PIC) (Sigma, P8340) diluted at 1:100. Next the lysates were threefold frozen-thawed and centrifuged at 10000 x *g* for 10 minutes at 4°C; supernatants were stored at -80°C.

Western blotting analysis

Protein concentration in cellular and tissue extracts was determined by the standard Bradford procedure (Bradford 1976). Samples of identical protein amount (30 μg) were separated by 10% polyacrylamide gel electrophoresis in the presence of sodium dodecylsulfate (SDS-PAGE) according to Laemmli (1970), as a molecular mass marker served PageRuler™ Prestained Protein Ladder (Fermentas). This was followed by transfer to nitrocellulose membrane, using the procedure described elsewhere (Towbin et al. 1979). Monoclonal, rabbit anti-gelsolin antibodies (Abcam, clone EPR1942) at dilution 1:20000; monoclonal, mouse anti-gelsolin antibodies (Sigma, clone GS-2C4) at dilution 1:2000 and polyclonal goat anti-gelsolin antibodies (Santa Cruz, clone C-20) at dilution 1:500 were used to visualize gelsolin band on nitrocellulose. Secondary antibodies conjugated to horseradish peroxidase (HRP) were applied according to the manufacturer's protocols (Cell Signaling). Immunoblots were developed using the Western blotting Luminol Reagent (Santa Cruz Biotechnology), photos of blots were taken with ChemiDoc™ MP System (Bio-Rad) and analyzed using densitometric analysis in ImageLab 4.0 software (Bio-Rad). In the case of staining with Ponceau S, the membranes after transfer and prior to blocking step were immersed in 0.2% Ponceau S for 10 min. The photos of stained membranes were taken with ChemiDoc™ MP System (Bio-Rad).

Immunohistochemistry

Whole embryos at stage HH33 and parts of embryos at stage HH36 were collected and fixed in 4% formaldehyde (FA) for at least 24 h. Next samples were dehydrated and embedded in Paraplast® (Sigma), 7 μm thick sections were cut with the help of a rotation microtome (Leitz) and mounted on SuperFrost Plus microscope slides (Menzel-Gläser). FA-fixed and paraffin-embedded sections of chicken embryos were deparaffinized in xylen and subsequently rehydrated by a standard procedure. Antigen retrival was performed by boiling slides in citrate buffer (10 mM Na-citrate pH 6.0, 0.05% Tween 20) for 20 min. Endogenous peroxidase activity was blocked according to EnVision™ System-HRP (AEC)$^+$ kit manufacturer's protocol (DAKO). Sections were blocked for 1 h with 3% goat serum in 1% bovine serum albumin (BSA) in 50 mM Tris pH 7.6. Following the slides were incubated overnight at 4°C with primary rabbit anti-gelsolin antibodies (Abcam, clone EPR1942) diluted at 1:500. As a negative control normal rabbit antibodies were used. DAKO EnVision™ System-HRP (AEC)$^+$

kit was used to visualize the sites recognized by primary antibodies. The reaction was developed with 3-amino-9 ethylocarbazol (AEC) for 10 min. Next the sections were counterstained with Mayer's hematoxylin solution (Sigma) for 10 min to visualize cell nuclei. Photos at lower magnifications were taken using Olympus SZ61 stereo microscope and photos at higher magnification were taken using Olympus FV500 microscope.

References

Bradford MM (1976) A rapid and sensitive method for the quantitation of microgram quantities of protein utilizing the principle of protein-dye binding. Anal Biochem 72:248–54.

Choe H, Burtnick LD, Mejillano M, et al. (2002) The Calcium Activation of Gelsolin: Insights from the 3Å Structure of the G4–G6/Actin Complex. J Mol Biol 324:691–702. doi: 10.1016/S0022-2836(02)01131-2

Katoh K, Kuma K, Toh H, Miyata T (2005) MAFFT version 5: improvement in accuracy of multiple sequence alignment. Nucleic Acids Res 33:511–8. doi: 10.1093/nar/gki198

Katoh K, Standley DM (2013) MAFFT multiple sequence alignment software version 7: improvements in performance and usability. Mol Biol Evol 30:772–80. doi: 10.1093/molbev/mst010

LAEMMLI UK (1970) Cleavage of Structural Proteins during the Assembly of the Head of Bacteriophage T4. Nature 227:680–685. doi: 10.1038/227680a0

Müller M, Mazur AJ, Behrmann E, et al. (2012) Functional characterization of the human α-cardiac actin mutations Y166C and M305L involved in hypertrophic cardiomyopathy. Cell Mol Life Sci 69:3457–79. doi: 10.1007/s00018-012-1030-5

Nag S, Ma Q, Wang H, et al. (2009) Ca2+ binding by domain 2 plays a critical role in the activation and stabilization of gelsolin. Proc Natl Acad Sci U S A 106:13713–8. doi: 10.1073/pnas.0812374106

Towbin H, Staehelin T, Gordon J (1979) Electrophoretic transfer of proteins from polyacrylamide gels to nitrocellulose sheets: procedure and some applications. Proc Natl Acad Sci U S A 76:4350–4.

Figures

A

```
Hs_gelsolin a               MAPH-----------RPAPALLCALSLALCALSLPVRAATASRGASQAGAPQGRVPEARP
Gd_gelsolin Pubmed          MGKQGFGYIFLTIFCTMALKLNCVSSVSVAGLGYVVTAAV--------------VLSAVP
Gd_gelsolin sequencing      MGKQGFGYIFLTVFCTMALKLNCVSSVSVAGLGYVVTAAV--------------VLSAVP
                            *. :             *  * *. *:::..*.  * **.              * .* *

Hs_gelsolin a               NSMVVEHPEFLKAGKEPGLQIWRVEKFDLVPVPTNLYGDFFTGDAYVILKTVQLRNGNLQ
Gd_gelsolin Pubmed          VSM-VEHAEFSKAGKEPGLQIWRIEKFDLVPVPKNLYGDFFTGDSYLVLNTIRQRSGNLQ
Gd_gelsolin sequencing      VSM-VEHAEFSKAGKEPGLQIWRIEKFDLVPVPKNLYGDFFTGDSYLVLNTIKQRSGNLQ
                            ** ***.** ************:*********.**********:*::*:*:: *.****

Hs_gelsolin a               YDLHYWLGNECSQDESGAAAIFTVQLDDYLNGRAVQHREVQGFESATFLGYFKSGLKYKK
Gd_gelsolin Pubmed          YDLHFWLGDESSQDERGAAAIFTVQMDDYLQGKAVQHREVQGHESSTFLGYFKSGIKYKA
Gd_gelsolin sequencing      YDLHFWLGDESSQDERGAAAIFTVQMDDYLQGKAVQHREVQGHESSTFLGYFKSGIKYKA
                            ****:***:*.**** *********:****:*:*********.**:*********:***

Hs_gelsolin a               GGVASGFKHVVPNEVVVQRLFQVKGRRVVRATEVPVSWESFNNGDCFILDLGNNIHQWCG
Gd_gelsolin Pubmed          GGVASGFRHVVPNEVTVQRLLQVKGRRTVRATEVPVSWESFNTGDCFILDLGSNIYQWCG
Gd_gelsolin sequencing      GGVASGFRHVVPNEVTVQRLLQVKGRRTVRATEVPVSWESFNTGDCFILDLGSNIYQWCG
                            *******:*******.****:******.**************.*********.**:****

Hs_gelsolin a               SNSNRYERLKATQVSKGIRDNERSGRARVHVSEEGTEPEAMLQVLGPKPALPAGTEDTAK
Gd_gelsolin Pubmed          SNSNRQERLKATVLAKGIRDNEKNGRAKVFVSEEGAEREEMLQVLGPKPSLPQGASDDTK
Gd_gelsolin sequencing      SNSNRQERLKATVLAKGIRDNERNGRAKVFVSEEGAEREEMLQVLGPKPSLPQGASDDTK
                            ***** ****** ::*******:.***:*.*****:* * *********:** *:.* :*

Hs_gelsolin a               EDAANRKLAKLYKVSNGAGTMSVSLVADENPFAQGALKSEDCFILDHGKDGKIFVWKGKQ
Gd_gelsolin Pubmed          TDTANRKLAKLYKVSNGAGNMAVSLVADENPFSQAALNTEDCFILDHGTDGKIFVWKGRS
Gd_gelsolin sequencing      TDTANRKLAKLYKVSNGAGNMAVSLVADENPFSQAALNTEDCFILDHGTDGKIFVWKGRS
                            *:****************.*:**********:*.**::*********.*********:.

Hs_gelsolin a               ANTEERKAALKTASDFITKMDYPKQTQVSVLPEGGETPLFKQFFKNWRDPDQTDGLGLSY
Gd_gelsolin Pubmed          ANSDERKAALKTATDFIDKMGYPKHTQVQVLPESGETPLFKQFFKNWRDKDQTEGLGEAY
Gd_gelsolin sequencing      ANSDERKAALKTATDFIDKMGYPKHTQVQVLPESGETPLFKQFFKNWRDKDQTEGLGEAY
                            **::*********:*** **.***:***.****.*************** ***:*** :*

Hs_gelsolin a               LSSHIANVERVPFDAATLHTSTAMAAQHGMDDDGTGQKQIWRIEGSNKVPVDPATYGQFY
Gd_gelsolin Pubmed          ISGHVAKIEKVPFDAATLHTSRAMAAQHGMEDDGSGKKQIWRIEGSEKVPVDPATYGQFY
Gd_gelsolin sequencing      ISGHVAKIEKVPFDAATLHTSRAMAAQHGMEDDGSGKKQIWRIEGSEKVPVDPATYGQFY
                            :*.*:*::*:*********** ********:***:*:*********:*************

Hs_gelsolin a               GGDSYIIILYNYRHGGRQGQIIYNWQGAQSTQDEVAASAILTAQLDEELGGTPVQSRVVQG
Gd_gelsolin Pubmed          GGDSYIIILYDYRHAGKQGQIIYTWQGAHSTQDEIATSAFLTVQLDEELGGSPVQKRVVQG
Gd_gelsolin sequencing      GGDSYIIILYDYRHAGKQGQIIYTWQGAHSTQDEIATSAFLTVQLDEELGGSPVQKRVVQG
                            *********:***.*:******.****:*****:*:**:**.********:***.*****

Hs_gelsolin a               KEPAHLMSLFGGKPMIIYKGGTSREGGQTAPASTRLFQVRANSAGATRAVEVLPKAGALN
Gd_gelsolin Pubmed          KEPPHLMSMFGGKPLIVYKGGTSREGGQTTPAQTRLFQVRSSTSGATRAVELDPAASQLN
Gd_gelsolin sequencing      KEPPHLMSMFGGKPLIVYKGGTSREGGQTTPAQTRLFQVRSSTSGATRAVELDPAASQLN
                            ***.****:*****:*:************:**.*******:.::*******: * *. **

Hs_gelsolin a               SNDAFVLKTPSAAYLWVGTGASEAEKTGAQELLRVLRAQPVQVAEGSEPDGFWEALGGKA
Gd_gelsolin Pubmed          SNDAFVLKTPSAAYLWVGRGSNSAELSGAQELLKVLGARPVQVSEGREPDNFWVALGGKA
Gd_gelsolin sequencing      SNDAFVLKTPSAAYLWVGRGSNSAELSGAQELLKVLGARPVQVSEGREPDNFWVALGGKA
                            ***************** *:..** :******:** *:****:** ***.** ******

Hs_gelsolin a               AYRTSPRLKDKKMDAHPPRLFACSNKIGRFVIEEVPGELMQEDLATDDVMLLDTWDQVFV
Gd_gelsolin Pubmed          PYRTSPRLKDKKMDAYPPRLFACSNKSGRFTIEEVPGDLTQDDLATDDVMILDTWDQVFV
Gd_gelsolin sequencing      PYRTSPRLKDKKMDAHPPRLFACSNKSGRFTIEEVPGDLTQDDLATDDVMILDTWDQVFV
                            .**************:********** ***.******:* *:********:*********

Hs_gelsolin a               WVGKDSQEEEKTEALTSAKRYIETDPANRDRRTPITVVKQGFEPPSFVGWFLGWDDDYWS
Gd_gelsolin Pubmed          WIGKDAQEEEKTEALKSAKRYIETDPASRDKRTPVTLVKQGLEPPTFSGWFLGWDDDYWS
Gd_gelsolin sequencing      WIGKDAQEEEKTEALKSAKRYIETDPASRDKRTPVTLVKQGLEPPTFSGWFLGWDDDYWS
                            *:***:*********.**********.**:***:*:****:***:* ***********

Hs_gelsolin a               VDPLDRAMAELAA 782   M - beginning of gelsolin (isoform a in Hs)
Gd_gelsolin Pubmed          VDPLQRAMADVDV 778  [M] - beginning of gelsolin isoform b (Hs)
Gd_gelsolin sequencing      VDPLQRAMADVDV 778   V - amino acids differing from published
                            ****:****:: .            in Pubmed sequence
```

B

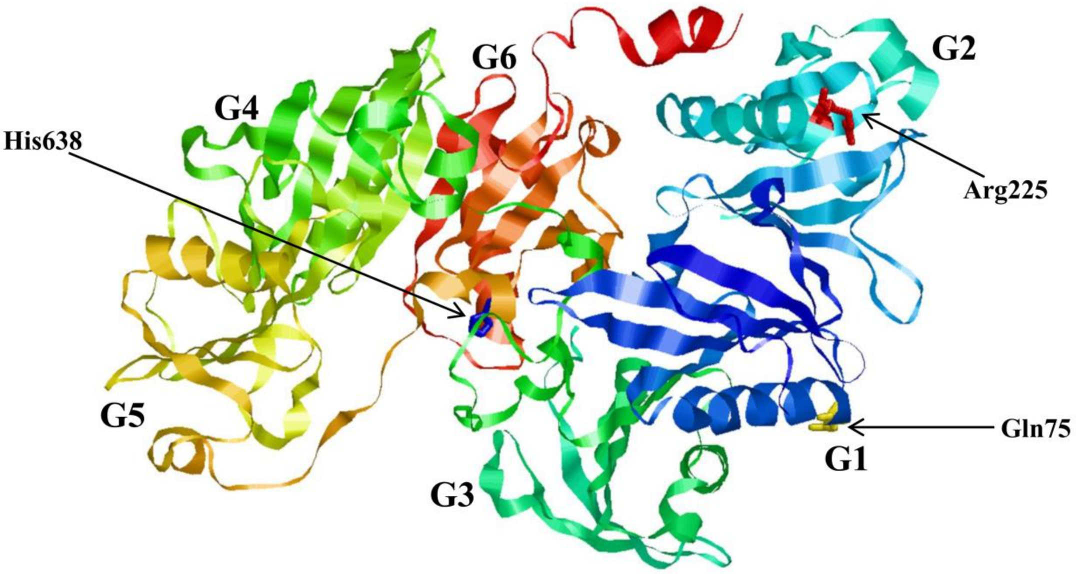

**Chicken gelsolin sequencing**	**Chicken gelsolin NP_990265.1**	**Human gelsolin NP_000168.1**	**Localization within gelsolin moiety**	**Position number in 3FFN structure**	**Function of amino acid in given position(Choe et al. 2002; Nag et al. 2009)**
Val13	Ile13	n/a	Signal peptide	n/a	No particular function
Lys98	Arg98	Gln102	Domain G1	Gln75	No particular function
Arg248	Lys248	Arg252	Domain G2	Arg225	No particular function
His661	Tyr661	His665	Linker between G5 and G6	His638	No particular function

Fig. S1 A: CLUSTAL format alignment of human (*Homo sapiens*) and chicken (*Gallus domesticus*) gelsolin primary structures found in Pubmed under following numbers: NP_000168.1 and NP_990265.1, respectively and gelsolin primary structure based on sequenced for this study DNA coding for gelsolin. The analysis was done with the help of the MAFFT program (Katoh et al. 2005; Katoh and Standley 2013). Note there are four amino acids (highlighted with grey boxes) differing from the chicken gelsolin amino acid sequence found under NP_990265.1 number (Pubmed). “*” means identical, “:” represents conserved substitutions, “.” means semi-conserved substitution. **B:** The structure of inactive gelsolin [PDB accession code: 3FFN (Nag et al. 2009)]. Visualization was performed with the help of the Swiss PDB Viewer. The table summarizes information about differing amino acids. Any of these residues possesses a particular function important for gelsolin functioning (Choe et al. 2002; Nag et al. 2009). n/a, not applicable

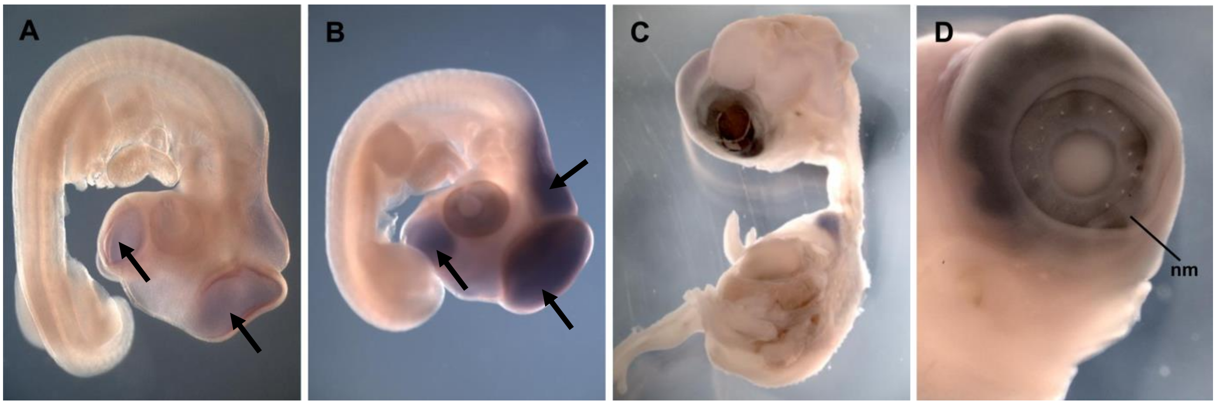

Fig. S2 Photos from *in situ* hybridization experiments with the sense probe for *GSN* transcript. **A:** Stage HH21 embryo. **B:** Stage HH25 embryo. **C:** Stage HH36 embryo cut longitudinal prior to the *in situ* hybridization. **D:** The head of the embryo at HH36-stage. Note the absence of any staining. The presence of staining within vesicles in the head region is due to the probe trapping (black arrows). nm, nictitating membrane.

A

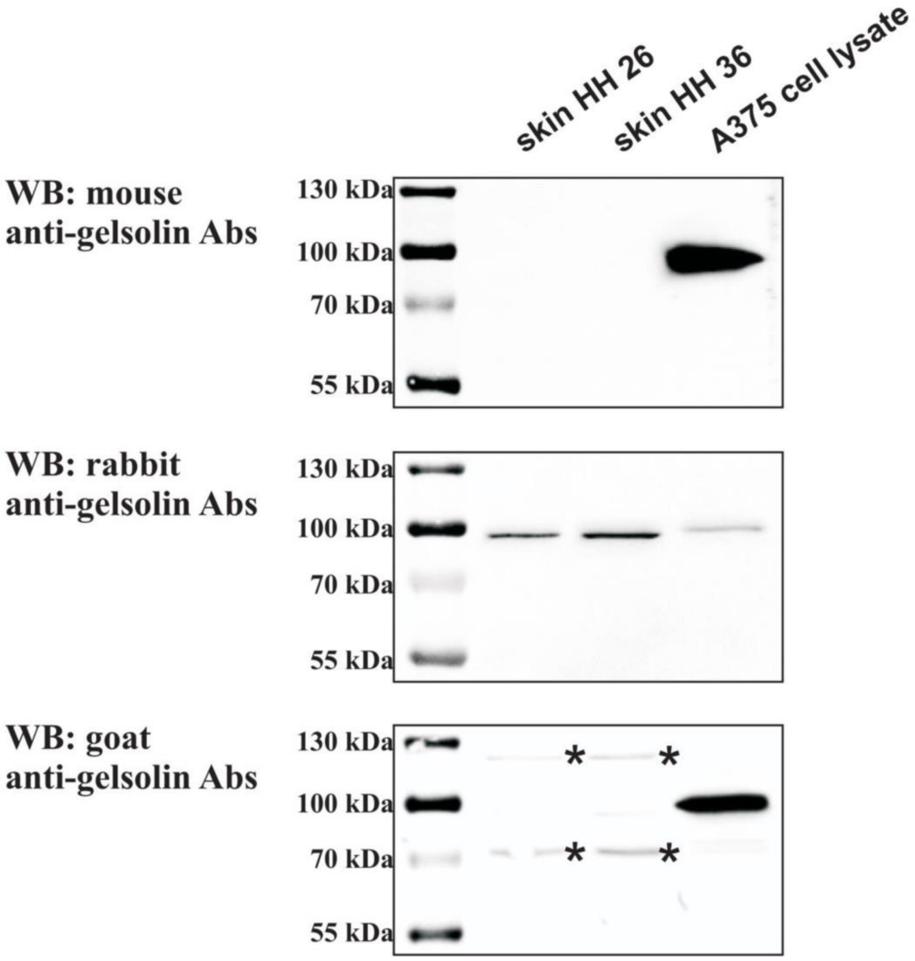

B

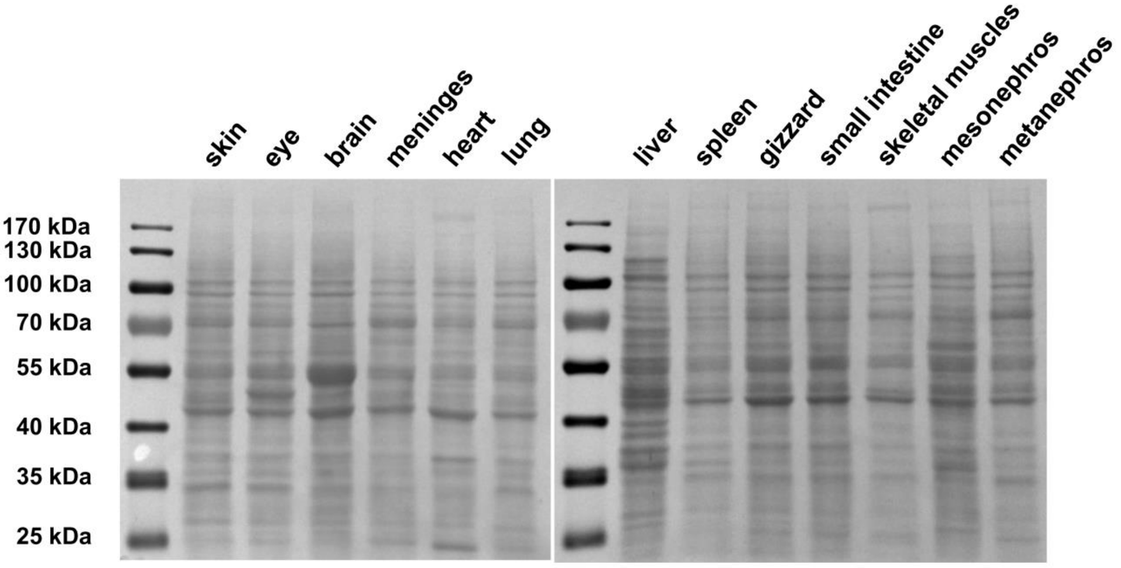

Fig. S3 A: Test of antibodies recognizing gelsolin. Lysates of dermis of 5- and 10-days old chicken embryos and cell lysate of human melanoma A375 cells were subjected to Western blotting analysis to identify antibodies recognizing chicken gelsolin. Note that only rabbit antibodies (Abcam, clone EPR1942) recognized chicken gelsolin. In the case of goat antibodies

(Santa Cruz, clone C-20) there were recognized some most probably unspecific bands at approx. 125 ad 72 kDa (stars) in lysates from chicken embryos skin. Mouse antibodies (Sigma, clone GS-2C4) did not recognize chicken gelsolin. **B:** Ponceau S staining of nitrocellulose membranes corresponding to Western blot analysis of tissues removed from chicken embryo at HH36 stage shown in Fig. 5B. 30 μg of protein was loaded on every line.

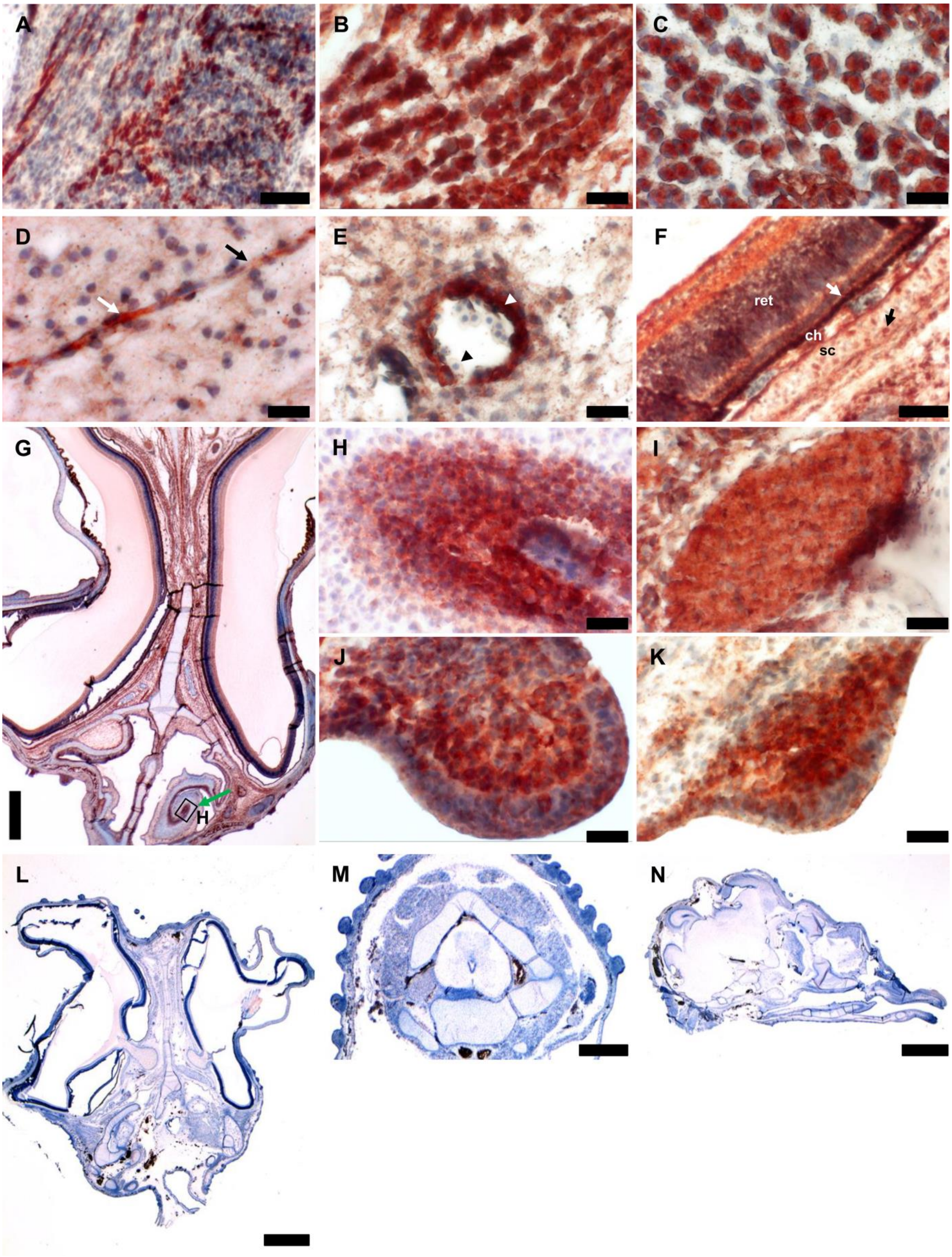

Fig. S4 Additional photos from immunohistochemical analysis of chicken embryo sections at stage HH36. **A:** Transverse section of neck muscles showing a strip-like pattern of gelsolin-positive cells. **B:** Frontal section of dorsal oblique muscle of eye. Note almost all muscle cells are stained for gelsolin. **C:** Transverse section of ventral oblique muscle of eye. All muscle

fibers are gelsolin-positive. **D:** Gelsolin-positive pericytes (white arrow) enwrapping a brain vessel (black arrow). **E:** Blood vessel within head. Smooth muscle cells building vessel wall are strongly gelsolin-positive (white arrowhead). Note the absence of gelsolin-positive blood cells (black arrowhead). **F:** Frontal section of retina, choroid and sclera of the eye. Note gelsolin is present in all mentioned layers including retinal pigment epithelium (short white arrow), however within scleral cartilage there were single gelsolin-positive cells (short black arrow). **G:** Transverse section of the head. Note strong signal of gelsolin-positive cells within the olfactory epithelium of the nasal conchae (green arrow). **H:** Higher magnification of section shown in G. **I:** Transverse section of an olfactory nerve. **J-K:** Feather buds located within the cranial dermis. **L-N:** Negative control stainings. There were used immunoglobulins from not immunized animals instead of antibodies directed against gelsolin. Sections were counterstained with Mayer's hematoxylin solution. Note the presence of staining only of cell nuclei. ch; choroid; ret, retina; sc, scleral cartilage. Scale bar: A, F 60 μm; B-E, H-K 25 μm; G, M 500 μm; L 1 mm; N 2 mm.

```
Gd_gelsolin      MGKQGFGYIFLTIFCTMALKLNCVSSVSVAGLGYVVTAAVVLSAVPVSMVEHAEFSKAGK
Gd_homogenin     MGKQGFGYIFLTIFCTMALKLNCVSSVSVAGLGYVVTAAVVLSAVPVSMVEHAEFSKAGK
                 ************************************************************

Gd_gelsolin      EPGLQIWRIEKFDLVPVPKNLYGDFFTGDSYLVLNTIRQRSGNLQYDLHFWLGDESSQDE
Gd_homogenin     EPGLQIWRIEKFDLVPVPKNLYGDFFTGDSYLVLNTIRQRSGNLQYDLHFWLGDESSQDE
                 ************************************************************

Gd_gelsolin      RGAAAIFTVQMDDYLQGKAVQHREVQGHESSTFLGYFKSGIKYKAGGVASGFRHVVPNEV
Gd_homogenin     RGAAAIFTVQMDDYLQGKAVQHREVQGHESSTFLGYFKSGIKYKAGGVASGFRHVVPNEV
                 ************************************************************

Gd_gelsolin      TVQRLLQVKGRRTVRATEVPVSWESFNTGDCFILDLGSNIYQWCGSNSNRQERLKATVLA
Gd_homogenin     TVQRLLQVKGRRTVRATEVPVSWESFNTGDCFILDLGSNIYQWCGSNSNRQERLKATVLA
                 ************************************************************

Gd_gelsolin      KGIRDNEKNGRAKVFVSEEGAEREEMLQVLGPKPSLPQGASDDTKTDTANRKLAKLYKVS
Gd_homogenin     KGIRDNEKNGRAKVFVSEEGAEREEMLQVLGPKPSLPQGASDDTKTDTANRKLAKLYKVS
                 ************************************************************

Gd_gelsolin      NGAGNMAVSLVADENPFSQAALNTEDCFILDHGTDGKIFVWKGRSANSDERKAALKTATD
Gd_homogenin     NGAGNMAVSLVADENPFSQAALNTEDCFILDHGTDGKIFVWKGRSANSDERKAALKTATD
                 ************************************************************

Gd_gelsolin      FIDKMGYPKHTQVQVLPESGETPLFKQFFKNWRDKDQTEGLGEAYISGHVAKIEKVPFDA
Gd_homogenin     FIDKMGYPKHTQVQVLPESGETPLFKQFFKNWRDKDQTEGLGEAYISGHVAKIEKVPFDA
                 ************************************************************

Gd_gelsolin      ATLHTSRAMAAQHGMEDDGSGKKQIWRIEGSEKVPVDPATYGQFYGGDSYIIILYDYRHAG
Gd_homogenin     ATLHTSRAMAAQHGMEDDGSGKKQIWRIEGSEKVPVDPATYGQFYGGDSYIIILYDYRHAG
                 ************************************************************

Gd_gelsolin      KQGQIIYTWQGAHSTQDEIATSAFLTVQLDEELGGSPVQKRVVQGKEPPHLMSMFGGKPL
Gd_homogenin     KQGQIIYTWQGAHSTQDEIATSAFLTVQLDEELGGSPVQKRVVQGKEPPHLMSMFGGKPL
                 ************************************************************

Gd_gelsolin      IVYKGGTSREGGQTTPAQTRLFQVRSSTSGATRAVELDPAASQLNSNDAFVLKTPSAAYL
Gd_homogenin     IVYKGGTSREGGQTTPAQTRLFQVRSSTSGATRAVELDPAASQLNSNDAFVLKTPSAAYL
                 ************************************************************

Gd_gelsolin      WVGRGSNSAELSGAQELLKVLGARPVQVSEGREPDNFWVALGGKAPYRTSPRLKDKKMDA
Gd_homogenin     WVGRGSNSAELSGAQELLKVLGARPVQVSEGREPDNFWVALGGKAPYRTSPRLKDKKMDA
                 ************************************************************

Gd_gelsolin      YPPRLFACSNKSGRFTIEEVPGDLTQDDLATDDVMILDTWDQVFVWIGKDAQEEEKTEAL
Gd_homogenin     YPPRLFACSNKSGRFTIEEVPGDLTQDDLATDDVMILDTWDQVFVWIGKDAQEEEKTEAL
                 ************************************************************

Gd_gelsolin      KSAKRYIETDPASRDKRTPVTLVKQGLEPPTFSGWFLGWDDDYWSVDPLQRAMADVDV 778
Gd_homogenin     KSAKRYIETDPASRDKRTPVTLVKQGLEPPTFSGWFLGWDDDYWSVDPLQRAMADVDV 778
                 **********************************************************
```

M – beggining of gelsolin

Fig. S5 A comparison of amino acid sequences of chicken gelsolin and chicken homogenin. CLUSTAL format alignment of chicken (*Gallus domesticus*) gelsolin (Pubmed: NP_990265.1) and chicken homogenin (Pubmed: AAC62928.1.) was done with the help of the MAFFT program (Katoh et al. 2005; Katoh and Standley 2013). Amino acid sequence of chicken gelsolin is in 100% identical to chicken homogenin. "*" means identical.
